# Controllability of dynamic double helices: quantitative analysis of the inversion of a screw-sense preference upon complexation†
†Electronic supplementary information (ESI) available: NMR, UV and CD spectroscopic data, energy-minimized structures and experimental details of new compound preparation. CCDC 1404043. For ESI and crystallographic data in CIF or other electronic format see DOI: 10.1039/c5sc02614h


**DOI:** 10.1039/c5sc02614h

**Published:** 2015-08-07

**Authors:** Ryo Katoono, Shunsuke Kawai, Kenshu Fujiwara, Takanori Suzuki

**Affiliations:** a Department of Chemistry , Faculty of Science , Hokkaido University , Sapporo 060-0810 , Japan . Email: katoono@sci.hokudai.ac.jp ; Fax: +81 11 706 2714 ; Tel: +81 11 706 3396

## Abstract

We describe a quantitative analysis of the complexation-induced inversion of a screw-sense preference based on a conformationally dynamic double-helix structure in a macrocycle. The macrocycle is composed of two twisting units (terephthalamide), which are spaced by two strands (1,3-bis(phenylethynyl)benzene), and is designed to generate a double-helix structure through twisting about a *C*_2_ axis in a conrotatory manner. The attachment of chiral auxiliaries to the twisting units induces a helical preference for a particular sense of (*M*)- or (*P*)-helicity through the intramolecular transmission of chirality to dynamic double helices. The twisting unit can also act as a binding site for capturing a guest molecule, and, in a complexed state, the preferred screw sense of the dynamic double-helix structure is reversed to exhibit the contrary preference. We quantitatively monitored the complexation-induced inversion of the screw-sense preference using ^1^H NMR spectroscopy, which enabled us to observe independently two species with (*M*)- or (*P*)-helicity in both the absence and presence of a guest molecule. Inversion of the screw-sense preference was induced upon complexation with an achiral guest as well as a chiral guest.

## Abstract 2

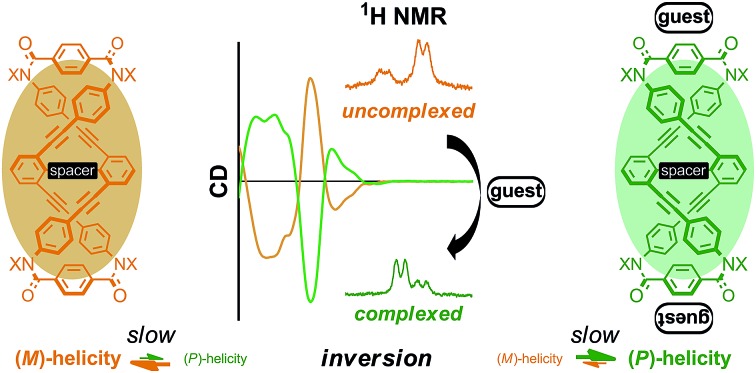
We analyzed quantitatively the complexation-induced inversion of a screw-sense preference of dynamic double helices with CD and ^1^H NMR spectroscopy.

## Introduction

Artificial double-helix structures^1^ have attracted a wide range of interest, including how they can be constructed as well as how they can be controlled to prefer a particular chiral sense. Supramolecular assemblies, based on hydrogen bonding, metal coordination or π–π stacking, of two oligomeric^2^ or polymeric^3^ strands with or without a chiral source have been extensively investigated. Alternative approaches to the construction of a double-helix structure have been based on the sequential connection of doubly helical fragments^4^ or twisting of a macrocycle.^5^–^7^ We were particularly interested in such a double helix with a well-defined molecular structure, which might be advantageous with respect to quantitative considerations. Chiroptical properties based on a double-helix structure have often been studied with stereochemically stable chiral macrocycles,^6^ in which only a particular sense of double helices was completely dominant and persistent. However, the control of dynamic chiroptical properties based on a double-helix structure with labile helical chirality^7^ has not been well studied. In this context, we studied a macrocycle with the purpose of developing such a rare dynamic system.

**Scheme 1:**
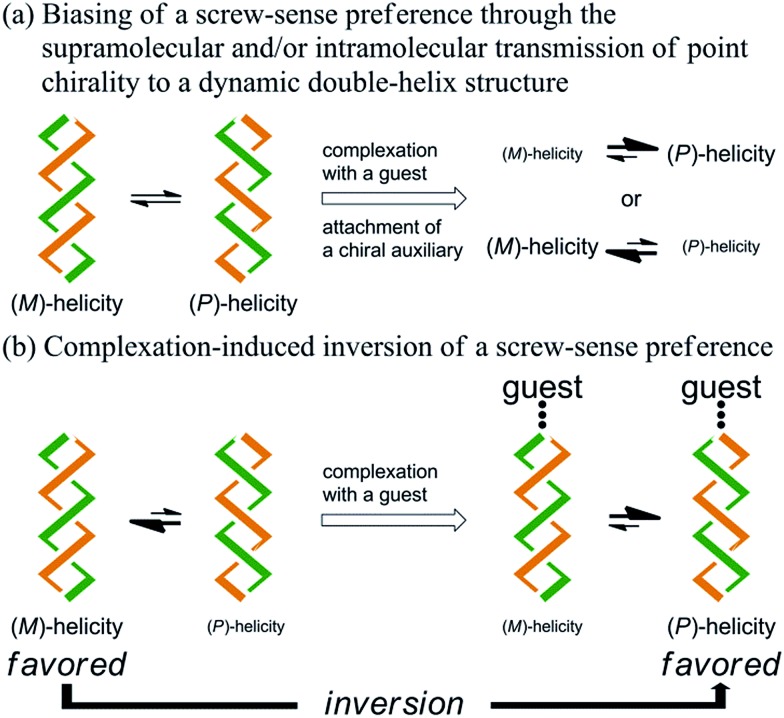


Here we report the successful control of a screw-sense preference based on a conformationally dynamic double-helix structure in a macrocycle, which includes not only simple biasing of the helical preference to a particular sense (Scheme 1a), but also inversion of the preferred screw sense to exhibit the contrary preference upon complexation with a guest molecule (Scheme 1b). In this article, we describe the complexation-induced inversion of a screw-sense preference of dynamic double helices through quantitative ^1^H NMR spectroscopy in addition to CD spectroscopy.

## Results and discussion

### Molecular design and preparation of macrocycles **1–3**

**Fig. 1:**
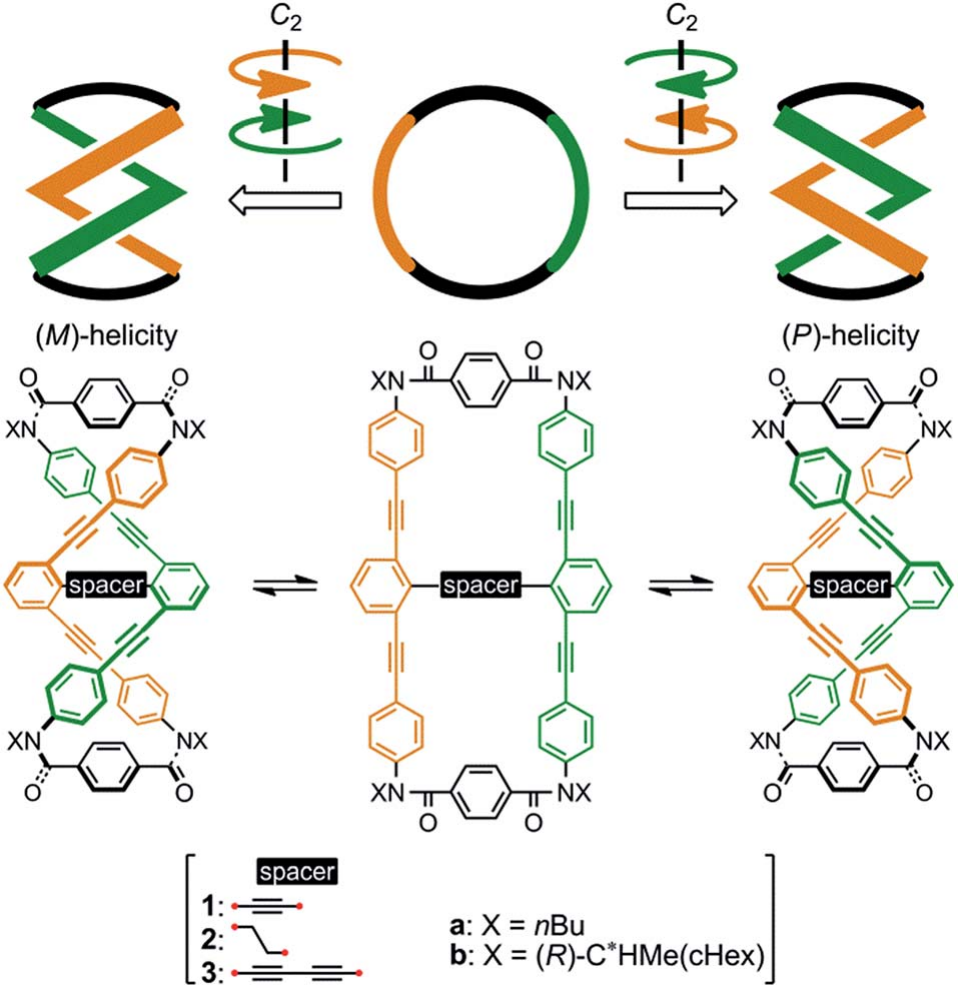
Chemical structures of macrocycles **1–3**, and dynamic interconversion between two double-helix structures with (*M*)- or (*P*)-helicity.

We designed a series of macrocycles **1–3**, in which a double-helix structure is generated through conrotatory twisting about a *C*_2_ axis toward (*M*)- or (*P*)-helicity (Fig. 1), as a new category of dynamic helical molecules based on terephthalamides.^8^,^9^ These macrocycles are composed of two twisting units (terephthalamide) that are spaced by two strands (1,3-bis(phenylethynyl)benzene), and are alternatively arranged to form a macrocyclic framework. Conrotatory twisting of the two amide groups in each twisting unit leads to two helical forms in the macrocycle, which can dynamically interconvert to each other through rotation about the C_C=O_–C_central_ bonds. The direction of the twisting can be controlled to prefer a particular sense through the transmission of chirality in a supramolecular^10^ and/or intramolecular^11^ manner upon complexation with a chiral ditopic^12^ guest at the two amide carbonyls (supramolecular) or the attachment of a chiral auxiliary to each amide nitrogen in the twisting units (intramolecular). We inserted a spacer between two strands so that they would be fixed and entangled to form a double-helix structure. We assumed that the motility of the double helices in the macrocyclic framework would be modulated by the spacing groups, for which we used ethynylene (**1**), ethylene (**2**), and butadiynylene (**3**) bonds. The substituent X on each amide nitrogen is an *n*-butyl group with no asymmetric element (**a**) or an (*R*)-C*HMe(cHex) group as a chiral auxiliary (**b**) in each macrocycle.

**Scheme 2:**
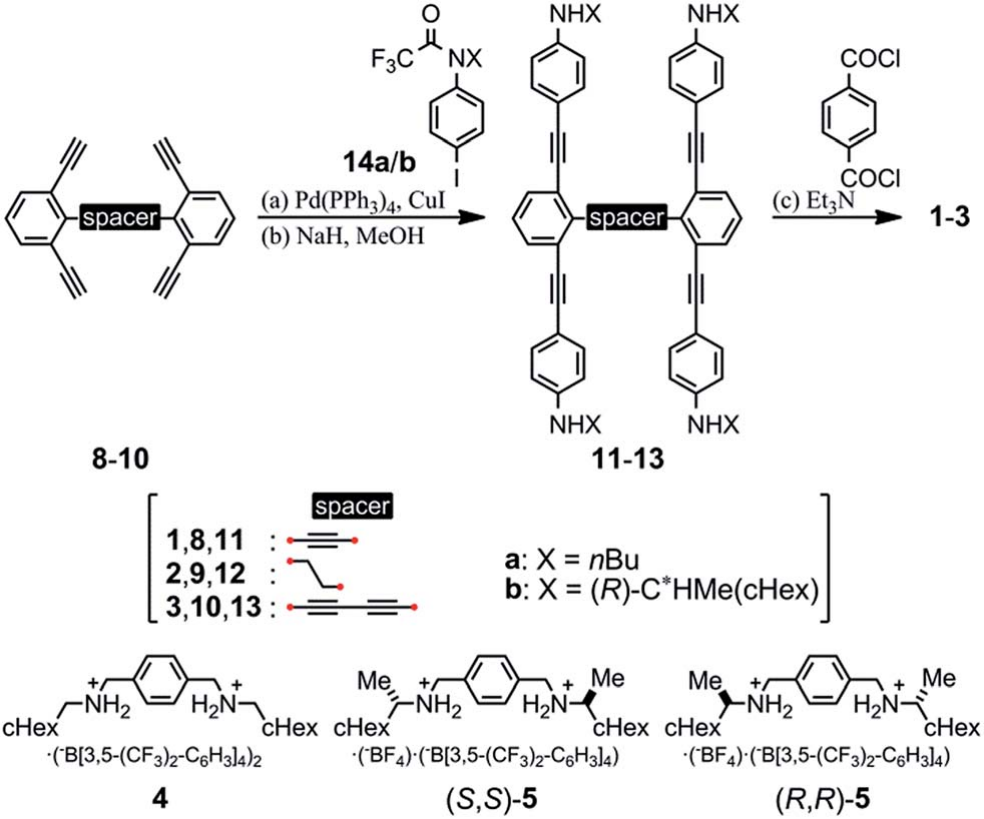
Preparation of macrocycles **1–3**. Yields: (a) 75%, (b) 54% for **11a**; (a) 76%, (b) 81% for **11b**; (a) 39%, (b) 96% for **12a**; (a) 82%, (b) 71% for **12b**; (a) 75%, (b) 98% for **13a**; (a) 87%, (b) 92% for **13b**; (c) 82% for **1a**; 18% for **1b**; 63% for **2a**; 39% for **2b**; 25% for **3a**; 38% for **3b**. Chemical structures of ditopic guests **4**, (*R*,*R*)-**5** and (*S*,*S*)-**5**.

We prepared macrocycles **1–3** through a four-fold condensation reaction of two equivalents of terephthaloyl chloride with the corresponding tetraanilines **11–13**, which were derived from the corresponding tetraynes **8–10** through a Sonogashira coupling reaction with *N*-trifluoroacetylated iodoaniline **14a** or (*R*)-**14b**,9*b* followed by deprotection of the trifluoroacetyl groups. The preparation of tetraynes **8–10** is summarized in Scheme S1.† We used diammonium salts **4**,9*c* (*R*,*R*)-**5** and (*S*,*S*)-**5**9*a* as ditopic guests to form a 1 : 2 complex with these macrocycles (Scheme 2).

### Molecular structures of macrocycles **1–3**

**Fig. 2:**
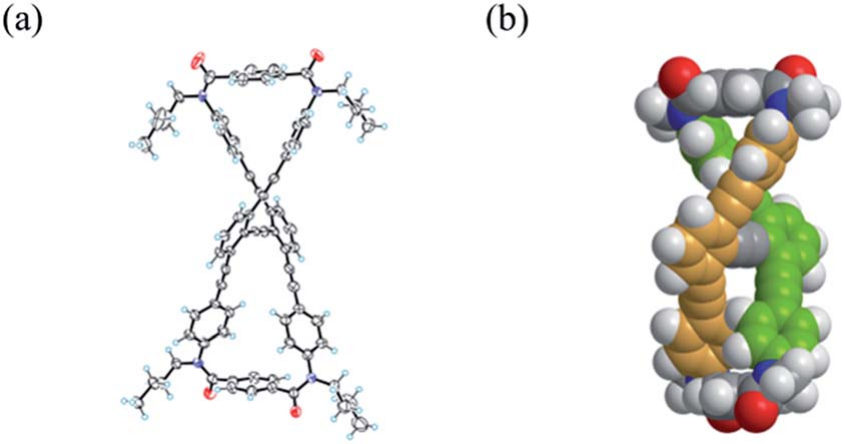
(a) X-ray structure of (*M*)-**1a** (*P*-1, *Z* = 2) in *rac*-**1a**·(benzene)_1.5_ crystal. Only one enantiomer is depicted. The solvent molecule was omitted for clarity. (b) Energy-minimized structure for **1a′** [NMe], obtained by a conformational search using the MacroModel software (v9.9 OPLS_2005, Monte Carlo Multiple Minimum method, non-solvated, 50 000 steps). Only one enantiomer is depicted.

We obtained a single crystal of **1a** that was appropriate for X-ray analysis, which revealed that the two amide groups of each terephthalamide were twisted in a conrotatory manner and led to global twisting of the molecule to give a double-helix structure in the macrocycle (Fig. 2a). In a crystal, two helical forms with (*M*)- or (*P*)-helicity appeared as a racemate. We found a similar conformation as the most energy-minimized structure in a conformational search for model **1a′** [NMe] (Fig. 2b), and expected that it would be the dominant conformation even in solution.

**Fig. 3:**
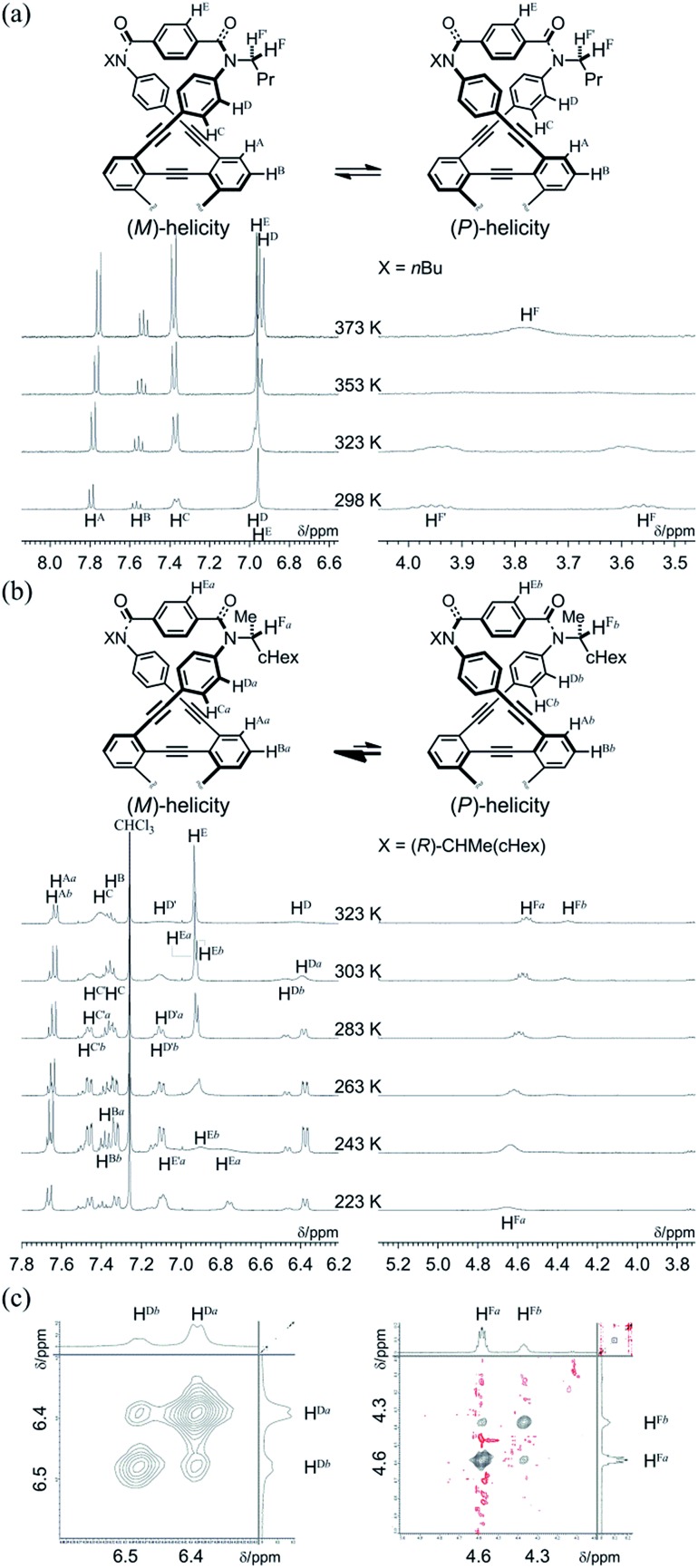
(a) Partial VT-^1^H NMR spectra (400 MHz) of **1a**, measured in dimethylsulfoxide-*d*_6_ at 293–383 K. (b) Partial VT-^1^H NMR spectra (400 MHz) of (*R*,*R*,*R*,*R*)-**1b**, measured in chloroform-*d* at 223–323 K. Two protons (H and H′, or H^a^ and H^b^) in a pair of split resonances are tentatively assigned. (c) Partial 2D-ROESY/EXSY spectra (600 MHz) of (*R*,*R*,*R*,*R*)-**1b**, measured in chloroform-*d* at 292 K (mixing time: 250 ms).

We investigated the dynamic structures of macrocycles in solution using ^1^H NMR spectroscopy at various temperatures (Fig. 3a). At room temperature, the spectrum of **1a** exhibited an anisochronous pair of signals for the methylene protons, which are closest to the amide nitrogen, and this indicated that they are in diastereotopic environments (H^F^ and H^F′^) in the molecule and that therefore the molecule is chiral on the NMR timescale at this temperature. The two signals coalesced at an elevated temperature (358 K in dimethylsulfoxide-*d*_6_). We assumed the averaging of two diastereotopic protons on the NMR-timescale to be the result of dynamic interconversion (enantiomerization)^13^ between two equivalent forms with (*M*)- or (*P*)-helicity in light of the above-mentioned conformational insights.^14^ Notably, there were almost no significant changes in the chemical shift during the VT measurements, although broadening and splitting were observed for some aromatic protons due to slow local rotations about the phenylene rings at lower temperatures. These results showed that the macrocyclic framework could mostly convert to the equivalent form.

The ^1^H NMR spectra of (*R*,*R*,*R*,*R*)-**1b**, measured in chloroform-*d* at 223–323 K, exhibited two non-equivalent sets of averaged resonances that were both assigned to *C*_2_ symmetry (Fig. 3b). The populations of two non-equivalent species changed with temperature, which indicated dynamic interconversion (stereoisomerization)11f–j,^15^ between two diastereomeric forms with (*M*)- or (*P*)-helicity in solution. This consideration was confirmed from the 2D-ROESY/EXSY spectrum of (*R*,*R*,*R*,*R*)-**1b**,^16^ measured at 292 K, in which we found cross-peaks that could be attributed to chemical exchange, not to NOE, for phenylene protons (H^Da^ and H^Db^) and methine protons (H^Fa^ and H^Fb^) (Fig. 3c).^15^,^17^ We considered the non-equivalent populations of the two diastereomeric species to be the result of a successful intramolecular transmission of point chirality (*R*) to dynamic double helices with (*M*)- or (*P*)-helicity in the macrocycle.^18^ The fact that we could observe two non-equivalent species independently at ambient temperature was important for quantitative monitoring of the complexation-induced inversion using ^1^H NMR spectroscopy (described later).

**Fig. 4:**
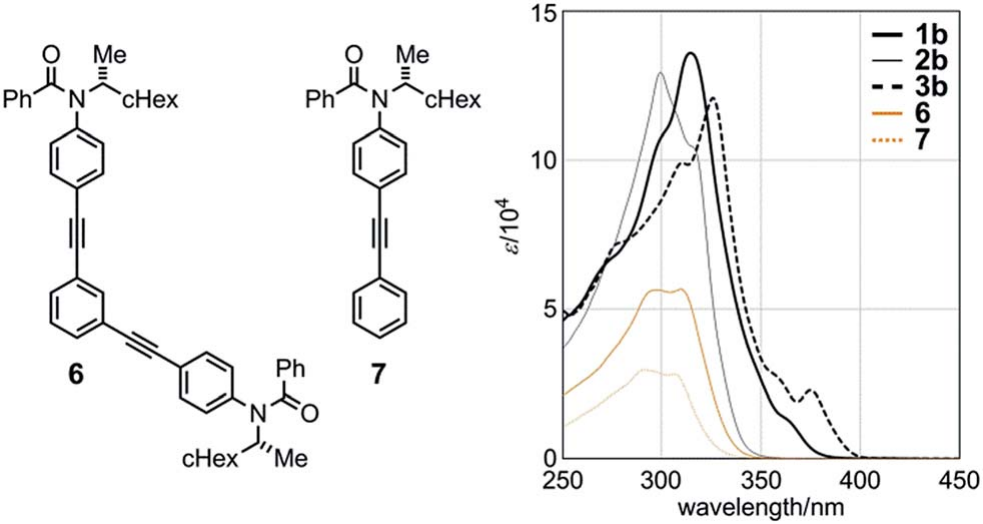
UV spectra of macrocycles **1b** (bold solid line), **2b** (thin solid line), and **3b** (bold dashed line), single-stranded substructure **6** (orange solid line), and diphenylacetylene **7** (orange dashed line), measured in CH_2_Cl_2_ at room temperature.

Macrocycle **2b** in dichloromethane showed absorption at 299 (log *ε* 5.11) and 317 (sh. 5.02) nm, which were similar in appearance to those in the spectra for single-stranded substructure **6** [298 (4.75) and 309 (4.75)] and diphenylacetylene derivative **7**9*e* [291 (4.47) and 307 (4.45)] (Fig. 4). The two strands in **2b** are spaced by saturated carbons and are present independently regarding the electronic structure to show a similarity in appearance to **6** and **7**. The molar absorptivity of **2b** was approximately twice that of **6**, which in turn was twice that of **7**. The absorption maximum and end in the spectra of **1b** and **3b** with a conjugated spacer were bathochromically shifted [*λ*_max_ 314 nm (log *ε* 5.13) for **1b** and 326 nm (5.08) for **3b**, end at around 400 nm] (Fig. 4). Similar observations were noted with **1a** [*λ*_max_ 315 (log *ε* 5.12)], **2a** [315 (sh. 4.98) and 299 (5.08)], and **3a** [325 (5.08)], which have an *n*-butyl group on each amide nitrogen, instead of (*R*)-C*HMe(cHex).

### Complexation of macrocyclic hosts **1a–3a** with chiral ditopic guests (*R*,*R*)-**5** and (*S*,*S*)-**5**

**Fig. 5:**
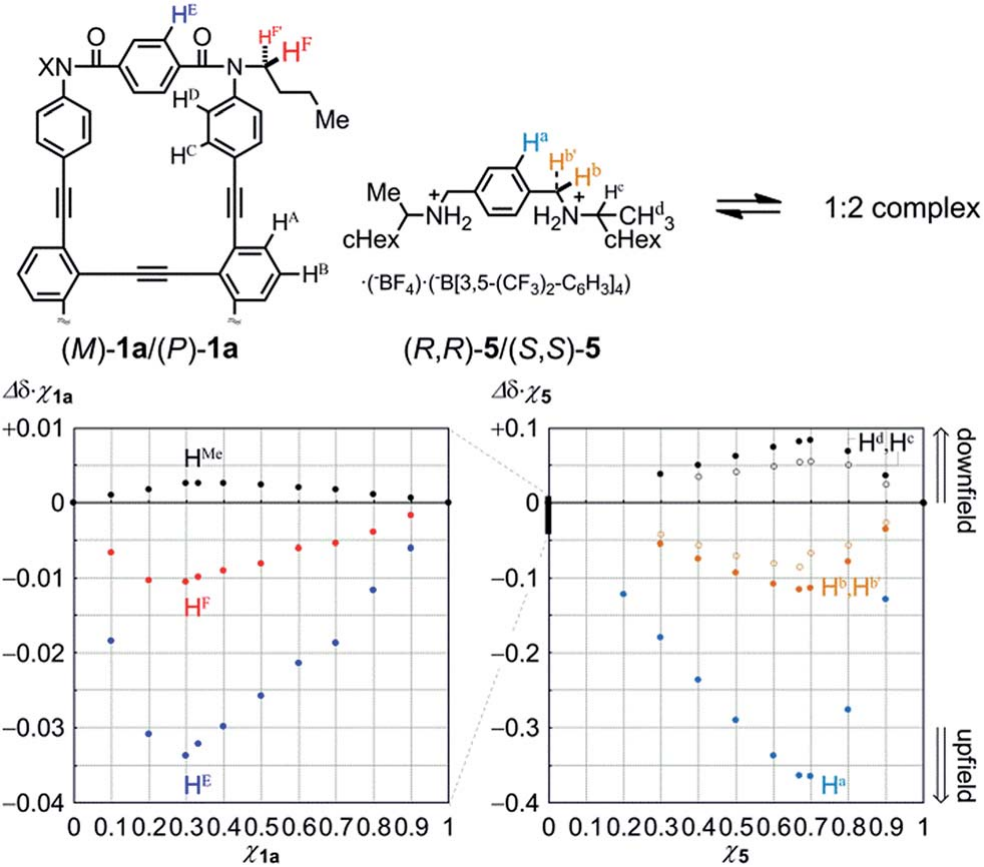
Job plots for the complexation of **1a** with an equivalent mixture of (*R*,*R*)-**5** and (*S*,*S*)-**5**, measured in CDCl_3_ at 303 K ([**1a**] + [**5**] = 2 mM), based on continuous changes in the chemical shifts induced for **1a** (left) and **5** (right).

We first examined the complexation of **1a** with a chiral ditopic guest **5**^19^ using ^1^H NMR spectroscopy. The spectra of **1a** in the presence of **5**, measured in chloroform-*d* at room temperature, showed upfield shifts for both the phenylene protons H^E^ in **1a** and the phenylene protons H^a^ in **5** (Fig. S4†), which indicated that the guest was captured at the two amide carbonyls in the twisting unit through the formation of hydrogen bonds. We confirmed that the stoichiometry was 1 : 2 using Job plots based on continuous changes in the chemical shifts induced for both **1a** and **5** (Fig. 5). There were marginal changes (Δ*δχ***_1a_** < 0.003) in the chemical shifts induced for other aromatic protons far from the binding site. This result showed that the host did not deform into any other structure during complexation. Similar upfield shifts with a slight difference in the chemical shift were induced when we used **2a** or **3a** as a host (Fig. S4†).

**Fig. 6:**
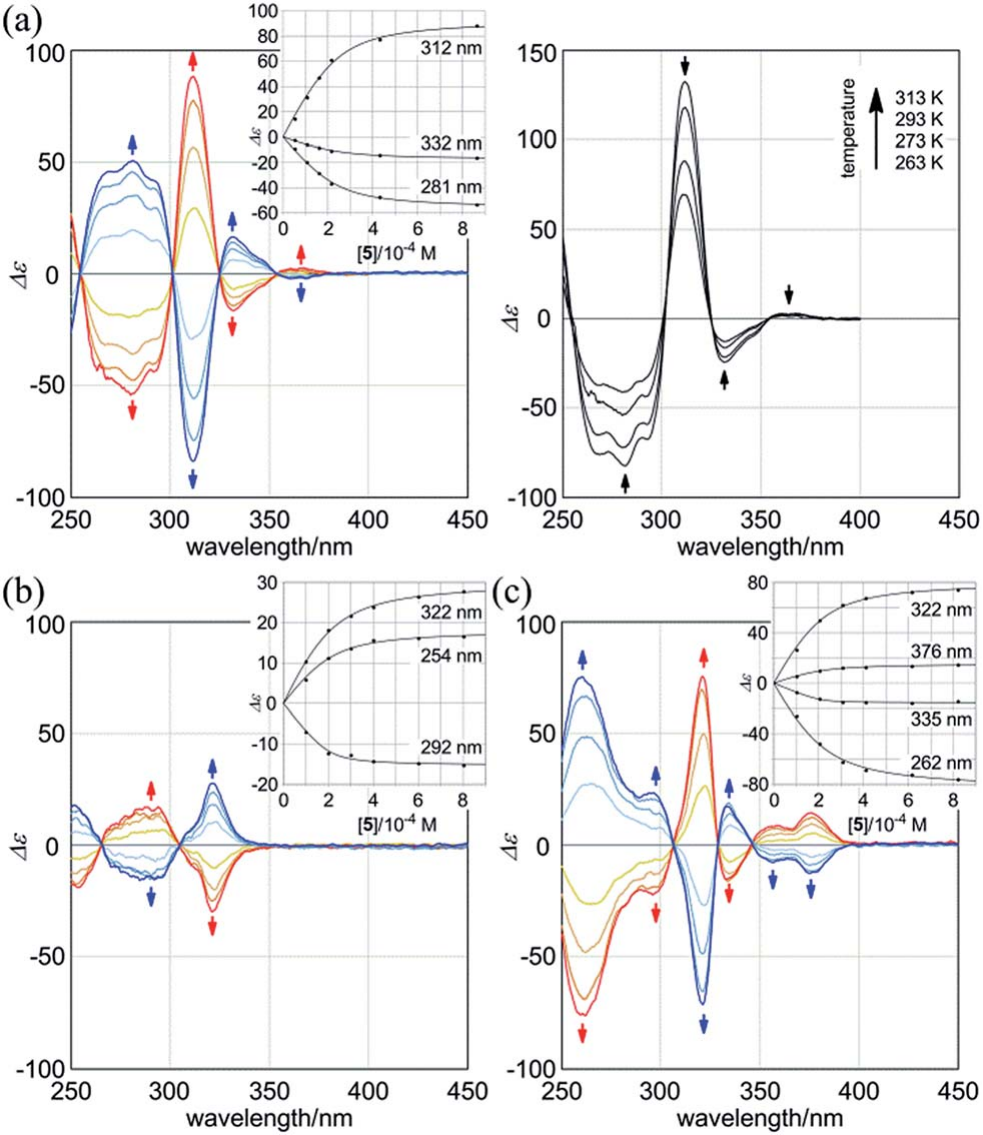
CD spectra of (a, left) **1a** (8.9–9.5 × 10^–5^ M), (b) **2a** (1.0 × 10^–4^ M), and (c) **3a** (1.0 × 10^–4^ M) in the presence of (*R*,*R*)-**5** (blue lines) or (*S*,*S*)-**5** (red lines) (1, 2, 4, and 8 equiv.), measured at 293 K [inset: plots of induced molar CD *versus* concentration of the guest added]. All spectra were measured in CH_2_Cl_2_ at 293 K. (a, right) VT-CD spectra of **1a** in the presence of (*S*,*S*)-**5** (8 equiv.), measured at 263–313 K.

Next, we monitored the 1 : 2 complexation of **1a** with the chiral ditopic guests (*R*,*R*)-**5** and (*S*,*S*)-**5** in dichloromethane to investigate the supramolecular transmission of point chirality to dynamic double helices. In the absence of any guest, we assumed an equivalent mixture of two enantiomeric forms with (*M*)- or (*P*)-helicity, since there is no asymmetric element in **1a** other than dynamic chirality. To a solution of **1a** (9.5 × 10^–5^ M) we gradually added up to eight equivalents of (*R*,*R*)-**5**. We found that compositive Cotton effects were continuously induced throughout the absorption region of **1a** (Fig. 6a, blue lines). When we used (*S*,*S*)-**5** instead of (*R*,*R*)-**5**, a set of mirror-imaged Cotton effects emerged (Fig. 6a, red lines). We considered these complexation-induced Cotton effects to be the result of a successful supramolecular transmission of point chirality in the guest to dynamic chirality in the host. In a complexed state, the host preferred a particular sense in response to the chirality in each guest. During the addition of a guest, there were almost no significant changes in the UV spectrum of **1a** (Fig. S5†), which indicated that the host mostly maintained the framework even in a complexed state. Similar observations were noted with **2a** and **3a** (Fig. 6b and c). Based on these titration experiments (see inset), we estimated binding constants (2 × 10^4^ M^–1^ for each binding site), and the details are summarized in Table S1.†

We further investigated the dynamic interconversion between two non-equivalent forms in a complexed state through VT measurements at lower and elevated temperatures (273 and 313 K). During the measurements in the presence of eight equivalents of (*S*,*S*)-**5**, molar CDs were enhanced with a decrease in temperature and attenuated with an increase in temperature, while the appearance of the spectrum did not change (Fig. 6a, right). This result showed that there was a dynamic equilibrium between two non-equivalent complexes [(*M*)-**1a**·(*S*,*S*)-**5**_2_ and (*P*)-**1a**·(*S*,*S*)-**5**_2_], and a particular form was favored over the other. These observations can be explained by one or both of the following considerations. First, some supramolecular assemblies such as hydrogen-bonded complexes driven by a gain of enthalpy are often favored at lower temperatures. Second, a preferred conformer is more favored at lower temperatures in an equilibrium between two non-equivalent conformers, as demonstrated in the VT ^1^H NMR measurements for (*R*,*R*,*R*,*R*)-**1b** and (*R*,*R*,*R*,*R*)-**2b**.

### Intramolecular transmission of point chirality to dynamic double helices in the macrocycle

**Fig. 7:**
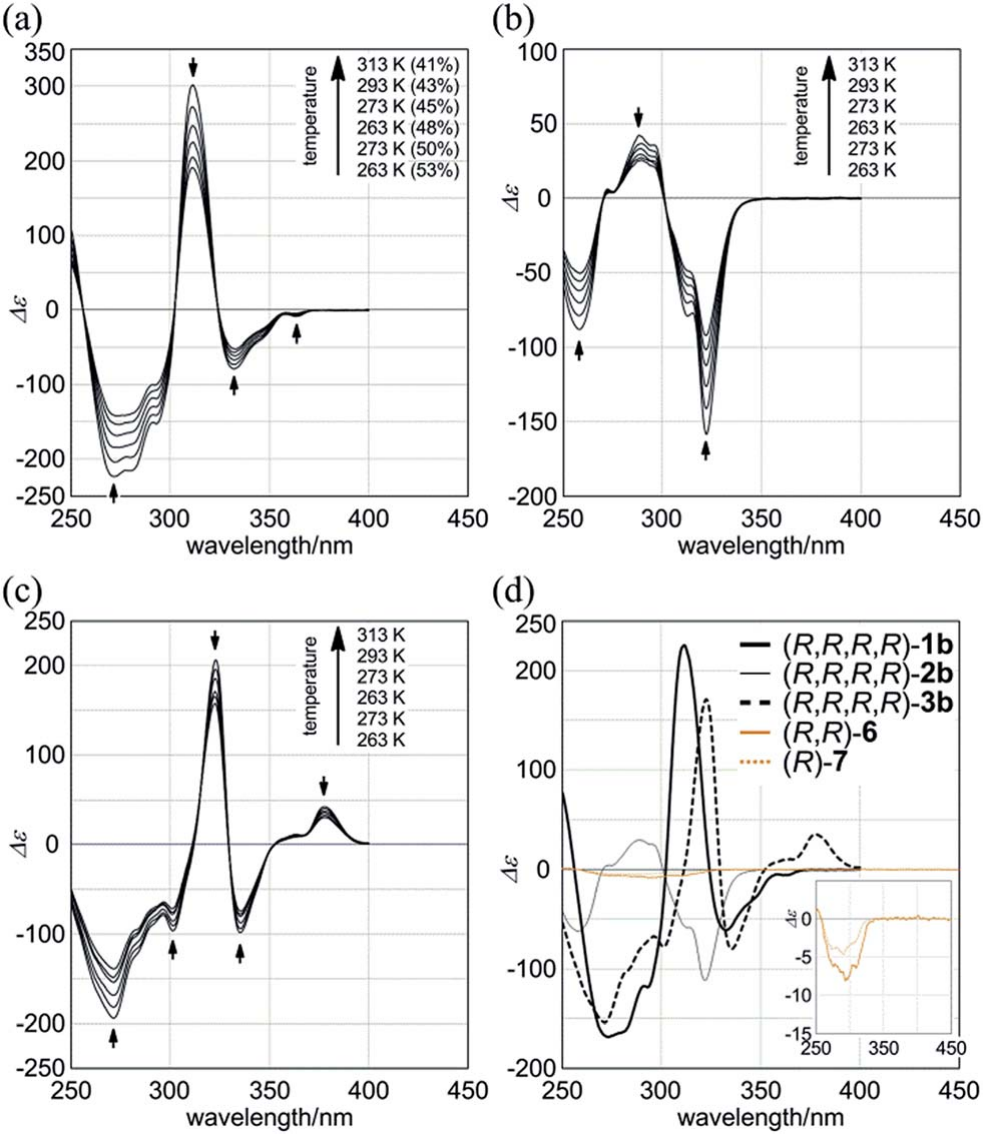
VT-CD spectra of (a) (*R*,*R*,*R*,*R*)-**1b**, (b) (*R*,*R*,*R*,*R*)-**2b**, and (c) (*R*,*R*,*R*,*R*)-**3b**, measured in CH_2_Cl_2_ at 263–313 K. (d) CD spectra of (*R*,*R*,*R*,*R*)-**1b**, (*R*,*R*,*R*,*R*)-**2b**, (*R*,*R*,*R*,*R*)-**3b**, single-stranded substructure (*R*,*R*)-**6**, and diphenylacetylene derivative (*R*)-**7**, measured in CH_2_Cl_2_ at 293 K. In (a), helical excess (%) is noted in parenthesis.

Macrocycles (*R*,*R*,*R*,*R*)-**1b**, (*R*,*R*,*R*,*R*)-**2b**, and (*R*,*R*,*R*,*R*)-**3b** showed compositive Cotton effects in each absorption region (Fig. 7). The spectral shape was similar to that seen for a complex of **1a**, **2a**, or **3a** with (*S*,*S*)-**6** (Fig. 6, red lines). These Cotton effects changed isosbestically with temperature. We considered the thermal response to be the result of dynamic interconversion between two diastereomeric forms with (*M*)- or (*P*)-helicity, and a particular form was favored over the other through the intramolecular transmission of internal chirality to dynamic chirality. For a solution of (*R*,*R*,*R*,*R*)-**1b**, we noted helical excess11g–j,^20^ at each temperature, which was estimated from a plot of ln *K versus* 1/*T* (Fig. S2a†). Single-stranded substructure (*R*,*R*)-**6** with the same internal chirality on each amide nitrogen showed Cotton effects that were totally different from those for macrocycles, but similar to those for (*R*)-**7** (Fig. 7d). We considered that these Cotton effects shown by (*R*,*R*)-**6** or (*R*)-**7** were due to a local chiral conformation around the internal chirality with no relation to a framework.

### Complexation-induced inversion of a screw-sense preference

Some dynamic helical molecules have been designed to undergo an inversion of helicity in response to a change in the environmental conditions^21^–^24^ or the addition of a guest molecule,^25^ and satisfy the following requirements: the dynamic molecule should have a helical preference for a particular sense in advance, and the preferred sense should be reversed to prefer the contrary sense in a different state. Here we demonstrate the complexation-induced inversion of a screw-sense preference based on dynamic double-helices in the macrocycles (*R*,*R*,*R*,*R*)-**1b**, (*R*,*R*,*R*,*R*)-**2b**, and (*R*,*R*,*R*,*R*)-**3b**, which were shown to have a preferred screw sense through the intramolecular transmission of chirality, as mentioned above.

**Scheme 3:**
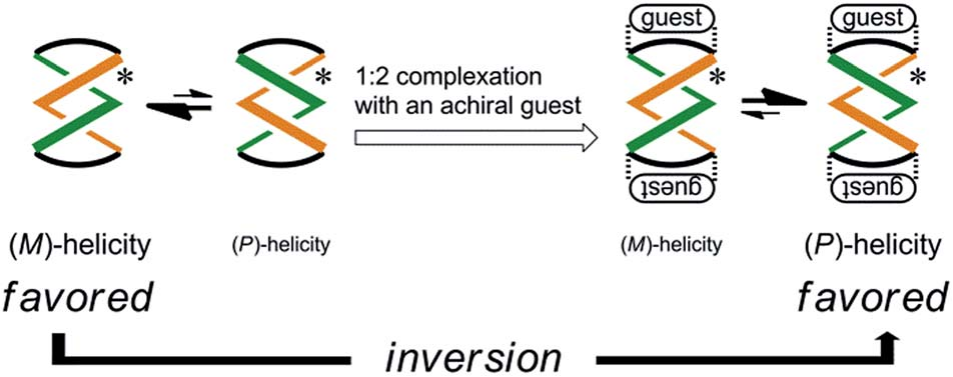
Inversion of a screw-sense preference upon complexation with an achiral guest.

First, the preferred screw sense of the dynamic double-helices was reversed to prefer the contrary sense upon 1 : 2 complexation with an achiral guest **4** (Scheme 3). This was the case for (*R*,*R*,*R*,*R*)-**1b** and (*R*,*R*,*R*,*R*)-**2b**. In both the uncomplexed and complexed states, the screw-sense preference was controlled through the intramolecular transmission of point chirality, since the achiral guest has no preference for a particular sense in the direction of twisting. The chiral auxiliaries associated with the host led to two contrary preferences before and after complexation.

**Scheme 4:**
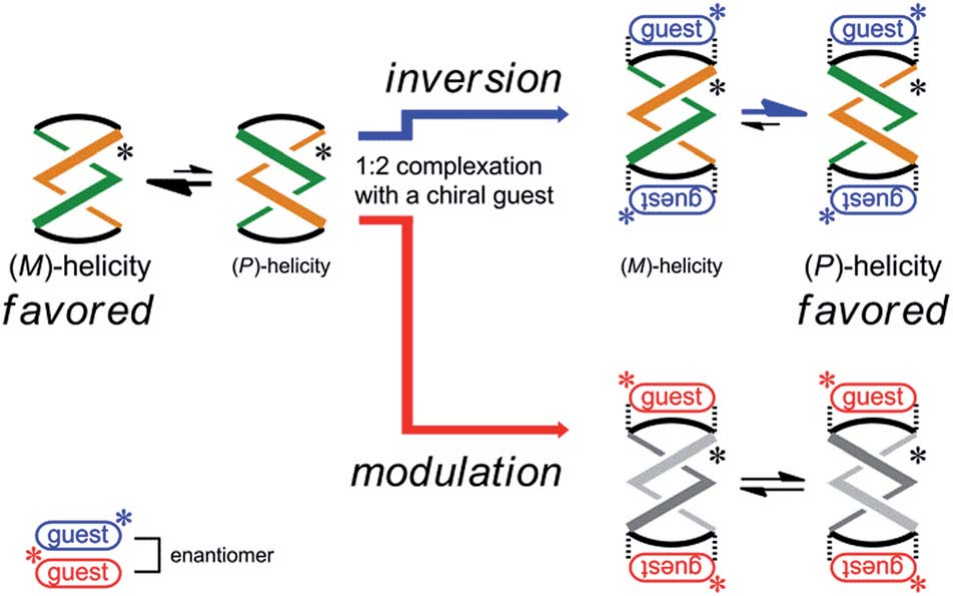
Stereospecific inversion of a screw-sense preference upon complexation with enantiomeric guests.

Next, the preferred sense was stereospecifically reversed upon 1 : 2 complexation with enantiomeric guests (*R*,*R*)-**5** and (*S*,*S*)-**5** (Scheme 4). This was the case for (*R*,*R*,*R*,*R*)-**3b**, which reversed the screw-sense preference only when it formed a complex with (*R*,*R*)-**5**. Alternatively, when we used the antipodal guest (*S*,*S*)-**5**, the host made the preference marginal even though a 1 : 2 complex was similarly formed. The details of these observations are described below.

**Fig. 8:**
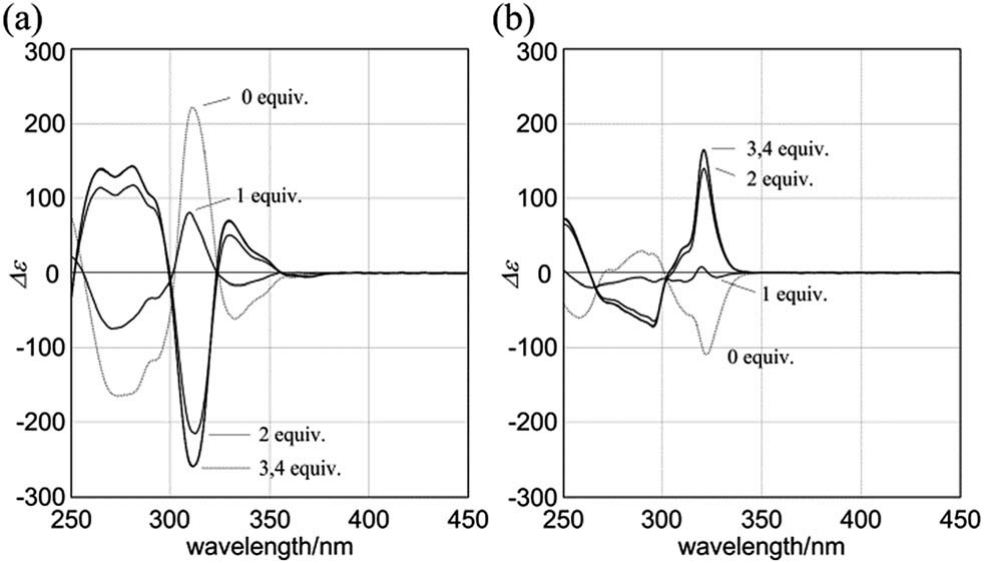
CD spectra of (a) (*R*,*R*,*R*,*R*)-**1b** (8.4 × 10^–5^ M) and (b) (*R*,*R*,*R*,*R*)-**2b** (1.0 × 10^–4^ M) in the presence of achiral ditopic guest **4** (solid lines) [0 (**1** only, dashed line), 1, 2, 3 and 4 equiv.]. All spectra were measured in CH_2_Cl_2_ at 293 K.

When (*R*,*R*,*R*,*R*)-**1b** and **4** were mixed in dichloromethane, the CD spectrum of (*R*,*R*,*R*,*R*)-**1b** changed continuously to show pseudo mirror-imaged Cotton effects (Fig. 8a). In both the absence and presence of **4**, the chiral auxiliaries associated with **1b** were dominantly transferred to dynamic double helices to exhibit contrary preferences. The two-way intramolecular transmission of point chirality was confirmed upon complexation even with chiral guests (*R*,*R*)-**5** and (*S*,*S*)-**5** (Fig. S6†). The intensity of induced Cotton effects in a complexed state was enhanced compared to that of the original effects whenever any ditopic guest was used, and this showed that the intramolecular transmission was more effective in a complexed state.^26^ Similar complexation-induced inversions of the screw-sense preference were noted with (*R*,*R*,*R*,*R*)-**2b** (Fig. 8b and Fig. S6†).

**Fig. 9:**
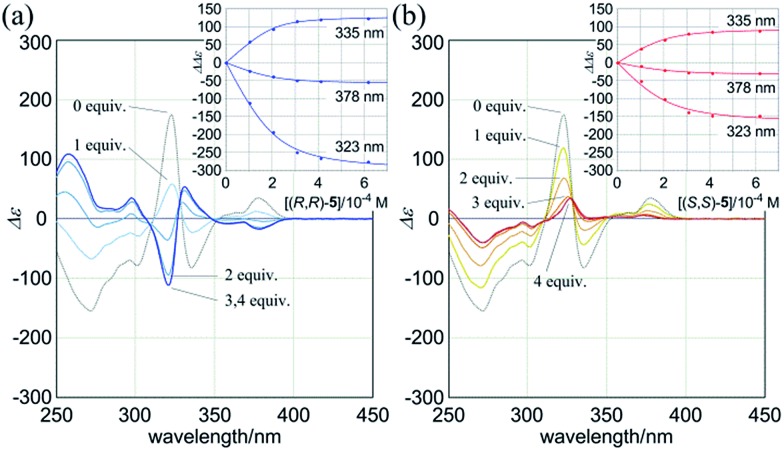
CD spectra of (*R*,*R*,*R*,*R*)-**3b** (1.0 × 10^–4^ M) in the presence of (a) (*R*,*R*)-**5** [0 (**3b** only, black dashed line), and 1–4 equiv. (blue lines)], (b) (*S*,*S*)-**5** [0 (**3b** only), and 1–4 equiv. (red lines)] [inset: plots of induced molar CD (ΔΔ*ε* = Δ*ε***_3b_**_·_**_5_** – Δ*ε***_3b_**) *versus* concentration of the added guest (0–6 equiv.)]. All spectra were measured in CH_2_Cl_2_ at 293 K.

Next, we mixed (*R*,*R*,*R*,*R*)-**3b** and (*R*,*R*)-**5** in dichloromethane. The CD spectrum of (*R*,*R*,*R*,*R*)-**3b** changed continuously to show pseudo mirror-imaged Cotton effects (Fig. 9a), although the intensity was reduced to some extent compared to the original effects, unlike in the above cases. The Cotton effects induced in a complexed state were similar to those seen in the spectrum of **3a** in the presence of (*R*,*R*)-**5**. Alternatively, when we added the antipodal (*S*,*S*)-**5** to a solution of (*R*,*R*,*R*,*R*)-**3b**, the original Cotton effects were continuously modulated to show marginal effects with a different appearance (Fig. 9b). Notably, the titration profiles (see inset) showed similar saturation points when up to six equivalents of each guest was added, and indicated that binding constants should be in a similar order (∼10^4^ M^–1^, Table S2†), although the ultimate shapes in each spectrum were different due to the local diastereomeric situation around asymmetric points. The modulated Cotton effects in a complexed state were considered to be the result of competition in the transmission of each chirality in the host and guest. These results indicated that the external chirality could contribute to determining the preference in a complexed state where the internal chirality was mostly negated but still present,^27^ and thus we identified the stereospecific inversion of a screw-sense preference based on a dynamic double-helix structure.

### Quantitative analysis of the complexation-induced inversion of a screw-sense preference based on ^1^H NMR spectroscopy

In most cases, an inversion of helicity has been demonstrated by the reversal of a sign of Cotton effects in CD spectroscopy.^21^–^25^ Alternatively, a quantitative analysis of the inversion of a screw-sense preference by the observation of two independent diastereomeric species that dynamically interconvert into each other is limited to only a few examples.^28^

**Fig. 10:**
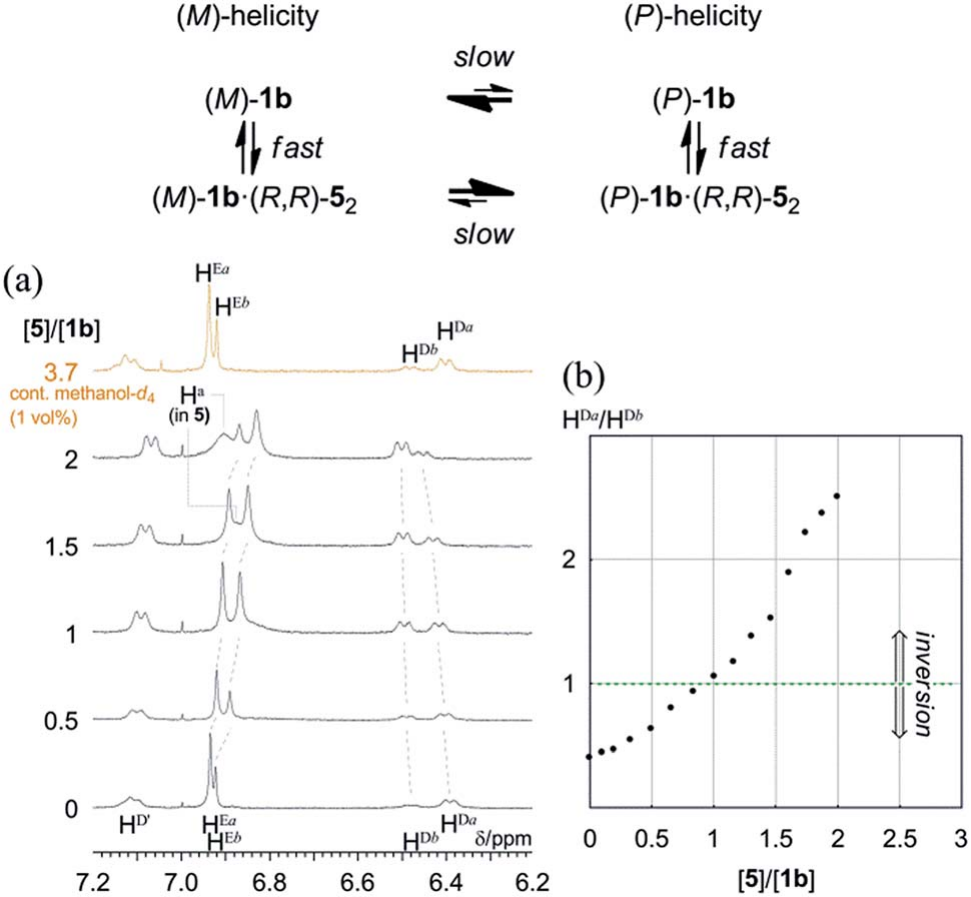
(a) ^1^H NMR spectra of (*R*,*R*,*R*,*R*)-**1b** (1 mM) in the presence of (*R*,*R*)-**5** (0–2 equiv., and 3.7 equiv. containing 1 vol% methanol-*d*_4_). All spectra were measured in CDCl_3_ at 293 K. (b) Plot of the ratio of the populations of two species with (*M*)- or (*P*)-helicity *versus* equivalents of **5** added, based on phenylene protons H^Da^ and H^Db^.

As mentioned above, only (*R*,*R*,*R*,*R*)-**1b** exhibited two non-equivalent species independently at room temperature by ^1^H NMR spectroscopy. In the spectrum, a particular set of averaged resonances was favored over the other. To a solution of (*R*,*R*,*R*,*R*)-**1b** (1 mM), (*R*,*R*)-**5** was gradually added as a ditopic guest that could render a complexed species dissolved under the complexation conditions. The spectra in the presence of (*R*,*R*)-**5** showed that the populations of two non-equivalent species (H^a^ and H^b^) continuously changed to ultimately be reversed (Fig. 10). Each set was assumed to be averaged between uncomplexed and complexed species due to fast exchange on the NMR-timescale. We confirmed that the original ratio was restored by the addition of methanol, which showed that the induced changes were due to the formation of hydrogen bonds.

## Conclusions

We have demonstrated the control of the screw-sense preference of dynamic double helices, which were generated in a macrocycle through conrotatory twisting about a *C*_2_ axis toward (*M*)- or (*P*)-helicity. The dynamic chirality was controlled to prefer a particular screw sense through the supramolecular transmission of external chirality upon 1 : 2 complexation with a chiral ditopic guest. The attachment of internal chirality also led to a screw-sense preference through the intramolecular transmission of chirality. Not only did we observe such simple biasing of the helical preference for a particular screw sense of a dynamic double-helix structure, we also demonstrated that the preferred screw sense was reversed upon 1 : 2 complexation with an achiral guest, as well as chiral guests. Through the systematic modulation of a spacing group, which was inserted between two strands, we identified multifarious inversions of a screw-sense preference upon complexation, which included inversion triggered by an achiral guest, and stereospecific inversion triggered only by a particular enantiomeric guest. Finally, we successfully monitored the inversion of the screw-sense preference in a quantitative manner using ^1^H NMR spectroscopy.

## Supplementary Material

Supplementary information

Crystal structure data
