# Supplementary material for: Controllability of dynamic double helices: quantitative analysis of the inversion of a screw-sense preference upon complexation
Source: Chem Sci. 2015 Aug 7;6(11):6592–600. doi: 10.1039/c5sc02614h (PMC5506618; doi:10.1039/c5sc02614h)
Supplement: Supplementary file 1 [file SC-006-C5SC02614H-s001.pdf]

Electronic Supplementary Material (ESI) for Chemical Science.
This journal is © The Royal Society of Chemistry 2015

Supplementary Information

Controllability of dynamic double helices: quantitative analysis of the inversion of a screw-sense preference upon complexation

Ryo Katoono,* Shunsuke Kawai, Kenshu Fujiwara and Takanori Suzuki

Department of Chemistry, Faculty of Science, Hokkaido University

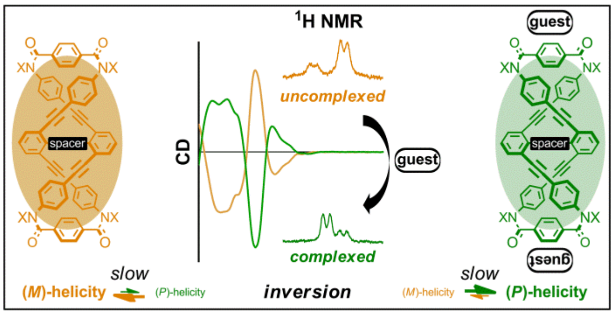

Contents

Supplementary Figures (Figure S1-S7)	S2-S6
Supplementary Tables (Table S1-S2)	S7
Supplementary Scheme (Scheme S1)	S8
Experimental	S8-18
^{1}H and ^{13}C NMR spectra of new compounds	S19-S34
References	S35

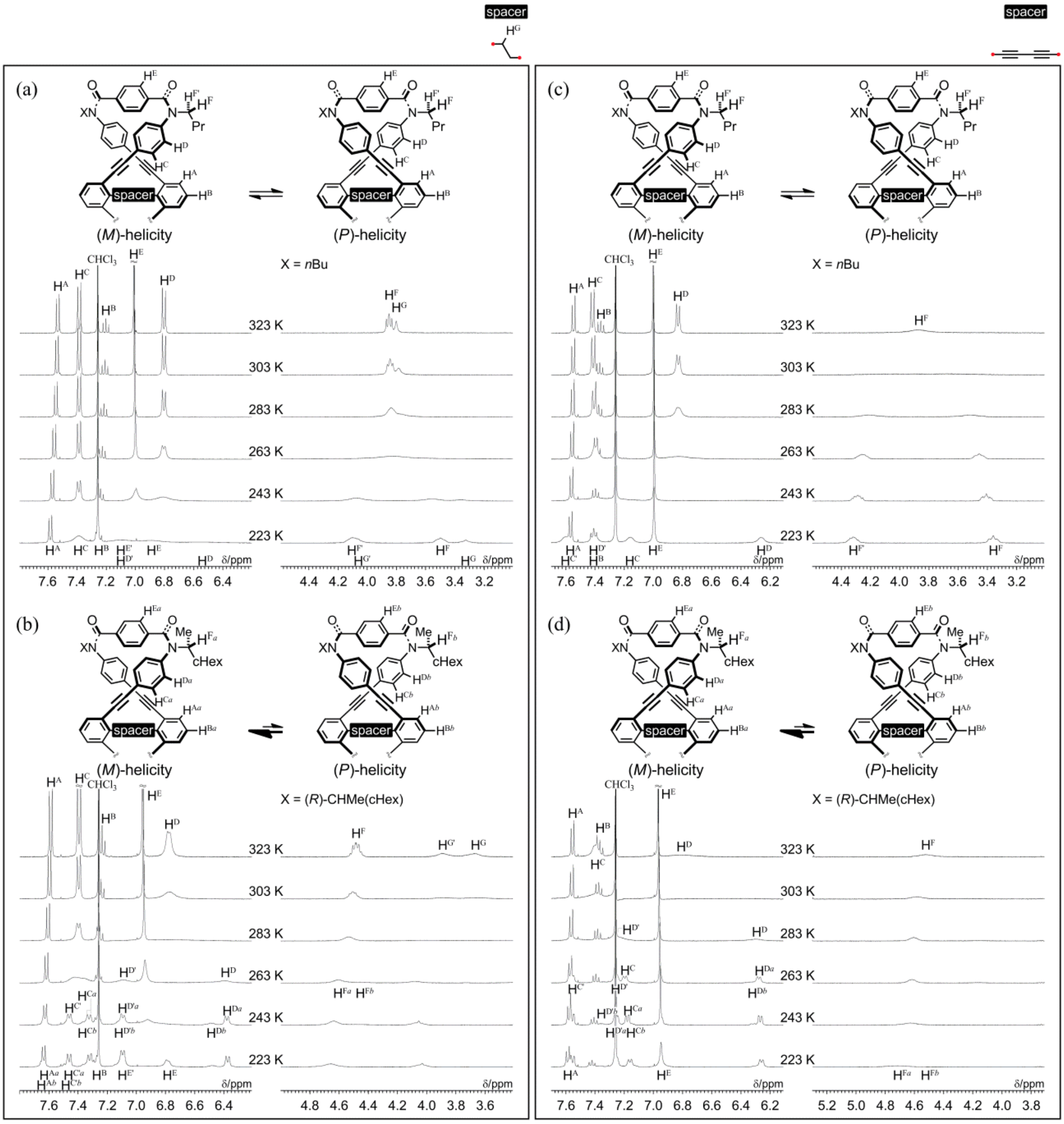

Figure S1. Partial VT-^{1}H NMR spectra (400 MHz) of (a) **2a**, (b) (R,R,R,R)-**2b**, (c) **3a**, and (d) (R,R,R,R)-**3b**, measured in $CDCl_3$ at 223-323 K. Two protons (H and H', or H^a and H^b) in a pair of split resonances are tentatively assigned.

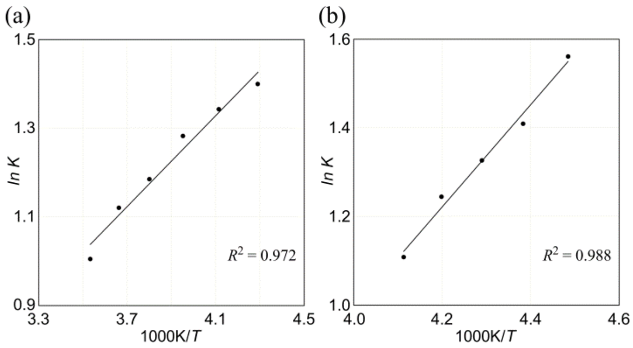

Figure S2. Plots of ln K versus $1/T$ for the dynamic interconversion between two forms with (*M*)- or (*P*)-helicity in a solution of (a) (*R*,*R*,*R*,*R*)-**1b** and (b) (*R*,*R*,*R*,*R*)-**2b**, based on values of integral for protons H^{Da} and H^{Db}, measured in $CDCl_3$ at (a) 233-283 K and (b) 223-243 K.

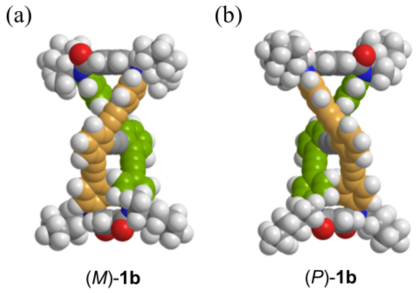

Figure S3A. Energy-minimized structures for (*R*,*R*,*R*,*R*)-**1b**, (a) (*M*)-**1b** (rel. 0 kJ mol^{-1}, most stable) and (b) (*P*)-**1b** (+2.65 kJ mol^{-1}), obtained by a conformational search using MacroModel software (v9.9 OPLS_2005, Monte Carlo Multiple Minimum method, non-solvated, 50 000 steps).

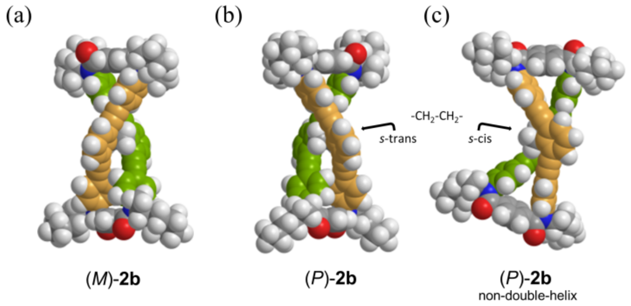

Figure S3B. Energy-minimized structures for (*R*,*R*,*R*,*R*)-**2b**, (a) (*M*)-**2b** (rel. 0 kJ mol^{-1}), (b) (*P*)-**2b** (+2.93 kJ mol^{-1}) and non-double-helical form (−1.57 kJ mol^{-1}, most stable), obtained by a conformational search using MacroModel software (v9.9 OPLS_2005, Monte Carlo Multiple Minimum method, non-solvated, 50 000 steps).

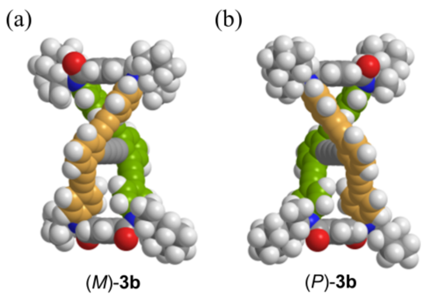

Figure S3C. Energy-minimized structures for (*R*,*R*,*R*,*R*)-**3b**, (a) (*M*)-**3b** (rel. 0 kJ mol^{-1}, most stable) and (b) (*P*)-**3b** (+1.48 kJ mol^{-1}), obtained by a conformational search using MacroModel software (v9.9 OPLS_2005, Monte Carlo Multiple Minimum method, non-solvated, 50 000 steps).

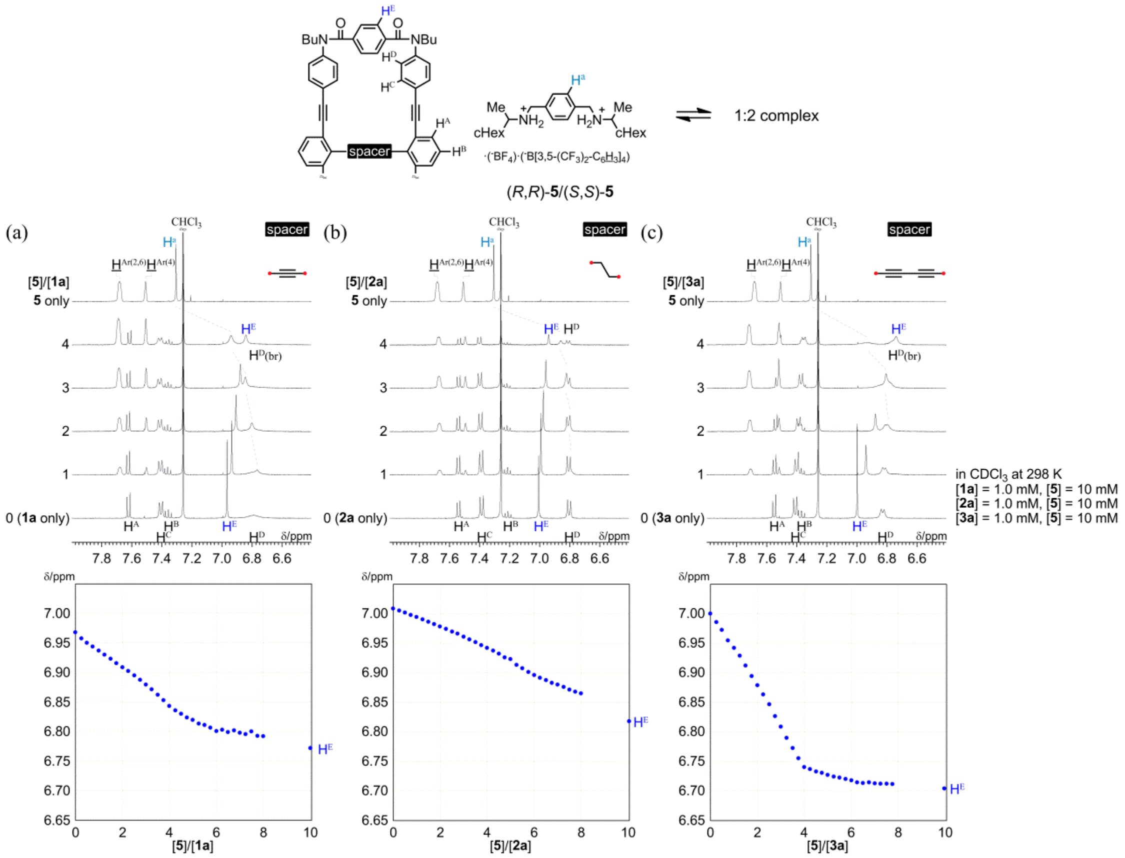

Figure S4. Partial ^{1}H NMR spectra (400 MHz) of (a) **1a**, (b) **2a**, and (c) **3a** in the presence of (*R*,*R*)-**5**/(*S*,*S*)-**5** (0-4 equiv.) (top), and plots of the chemical shift (δ/ppm) for the phenylene protons H^E versus equivalents of **5** ([**5**]/[**1a**], [**5**]/[**2a**], or [**5**]/[**3a**]) added (bottom).

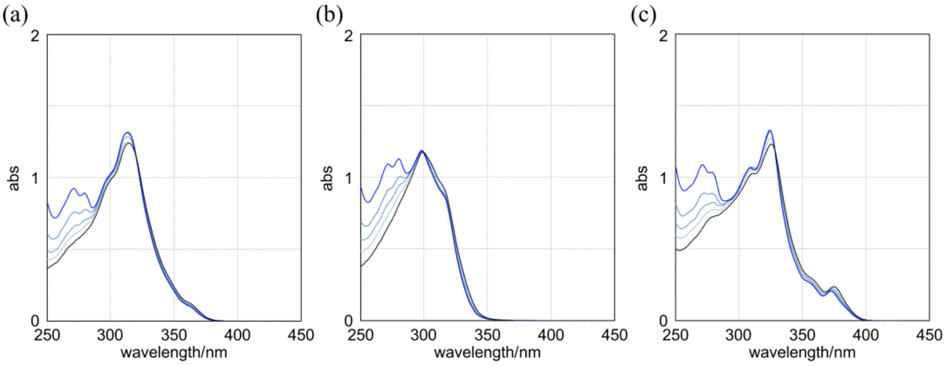

Figure S5. UV spectra of (a) **1a** (9.5×10^{-5} M), (b) **2a** (1.0×10^{-4} M), and (c) **3a** (1.0×10^{-4} M) in the presence of (*R*,*R*)-**5** (blue lines) [0 (**1a**, **2a**, or **3a** only, black line), 1, 2, 4, and 8 equiv.], measured in CH_2Cl_2 at room temperature.

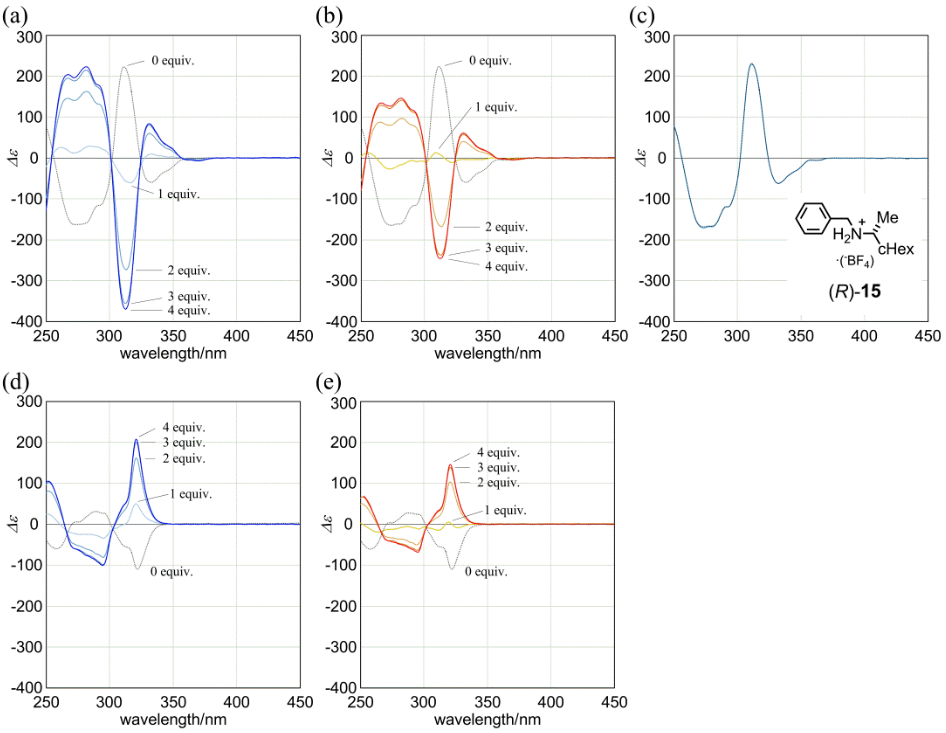

Figure S6. CD spectra of (a) (*R*,*R*,*R*,*R*)-**1b** (9.9×10^{-5} M) in the presence of (*R*,*R*)-**5** [0 (**1b** only, black dashed line), and 1-4 equiv. (blue lines)], (b) (*R*,*R*,*R*,*R*)-**1b** (1.0×10^{-4} M) in the presence of (*S*,*S*)-**5** [0 (**1b** only), and 1-4 equiv. (red lines)], (c) (*R*,*R*,*R*,*R*)-**1b** (9.2×10^{-5} M) in the presence of (*R*)-**15** [0 (**1b** only), and 8 equiv. (light blue line)], (d) (*R*,*R*,*R*,*R*)-**2b** (1.1×10^{-4} M) in the presence of (*R*,*R*)-**5** [0 (**2b** only, black dashed line), and 1-4 equiv. (blue lines)], and (e) (*R*,*R*,*R*,*R*)-**2b** (1.1×10^{-4} M) in the presence of (*S*,*S*)-**5** [0 (**2b** only), and 1-4 equiv. (red lines)]. All spectra were measured in CH_2Cl_2 at 293 K.

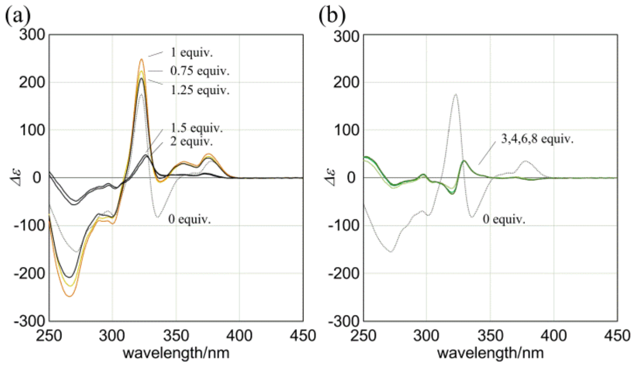

Figure S7. CD spectra of (*R*,*R*,*R*,*R*)-**3b** (1.1 × 10-4 M) in the presence of **4** (a) [0 (**3b** only), 0.75 and 1 equiv. (orange lines), and 1.25-2 equiv. (black solid lines)], and (b) [0 (**3b** only), and 3-8 equiv. (green lines)]. All spectra were measured in CH_2Cl_2 at 293 K.

Table S1. Binding constants,[a] obtained by a curve fitting method, based on CD titration experiments.

		(*R*,*R*)-**5** or (*S*,*S*)-**5**[b]			
[**1a**] = 1.16×10^{-4}/M		281 nm	312 nm	332 nm	
	K/M^{-1}	2.44×10^{4}	2.33×10^{4}	1.97×10^{4}	
	$\Delta\varepsilon_{max}$	-5.65×10^{1}	9.31×10^{1}	-1.75×10^{1}	
	χ^2	5.02×10^{0}	2.62×10^{1}	6.48×10^{-1}	
	R^2	9.98×10^{-1}	9.95×10^{-1}	9.96×10^{-1}	
[**2a**] = 1.00×10^{-4}/M		254 nm	292 nm	322 nm	
	K/M^{-1}	2.05×10^{4}	6.91×10^{4}	1.70×10^{4}	
	$\Delta\varepsilon_{max}$	1.81×10^{1}	-1.53×10^{1}	3.02×10^{1}	
	χ^2	9.67×10^{-1}	1.40×10^{0}	4.54×10^{-1}	
	R^2	9.92×10^{-1}	9.72×10^{-1}	9.98×10^{-1}	
[**3a**] = 1.03×10^{-4}/M		262 nm	322 nm	335 nm	376 nm
	K/M^{-1}	1.64×10^{4}	2.03×10^{4}	1.35×10^{5}	2.33×10^{4}
	$\Delta\varepsilon_{max}$	-8.38×10^{1}	8.06×10^{1}	-1.55×10^{1}	1.52×10^{1}
	χ^2	1.67×10^{1}	9.79×10^{0}	1.52×10^{0}	5.68×10^{-1}
	R^2	9.94×10^{-1}	9.97×10^{-1}	9.71×10^{-1}	9.90×10^{-1}

[a]Assume 1:2 complexation ($K_1 = K_2$). Doubled concentration (= [terephthalamide unit]) was used.
[b][(*R*,*R*)-**5**] or [(*S*,*S*)-**5**] = 0-8 equiv.

Table S2. Binding constants,[a] obtained by a curve fitting method, based on CD titration experiments.

		(*R*,*R*)-**5**[b]			(*S*,*S*)-**5**[b]		
[(*R*,*R*,*R*,*R*)-**3b**]= 1.03×10^{-4}/M^{-1}		323 nm	335 nm	378 nm	323 nm	335 nm	378 nm
	K/M^{-1}	2.62×10^{4}	5.23×10^{4}	3.31×10^{4}	2.11×10^{4}	3.28×10^{4}	2.11×10^{4}
	$\Delta\Delta\varepsilon_{max}$	-3.05×10^{2}	1.30×10^{2}	-5.88×10^{1}	-1.71×10^{2}	9.60×10^{1}	-3.42×10^{1}
	χ^2	1.51×10^{2}	1.82×10^{1}	4.45×10^{0}	2.67×10^{2}	9.48×10^{0}	1.22×10^{1}
	R^2	9.98×10^{-1}	9.98×10^{-1}	9.98×10^{-1}	9.87×10^{-1}	9.98×10^{-1}	9.85×10^{-1}

[a]Assume 1:2 complexation ($K_1 = K_2$). Doubled concentration (= [terephthalamide unit]) was used.
[b][(*R*,*R*)-**5**] or [(*S*,*S*)-**5**] = 0-6 equiv.

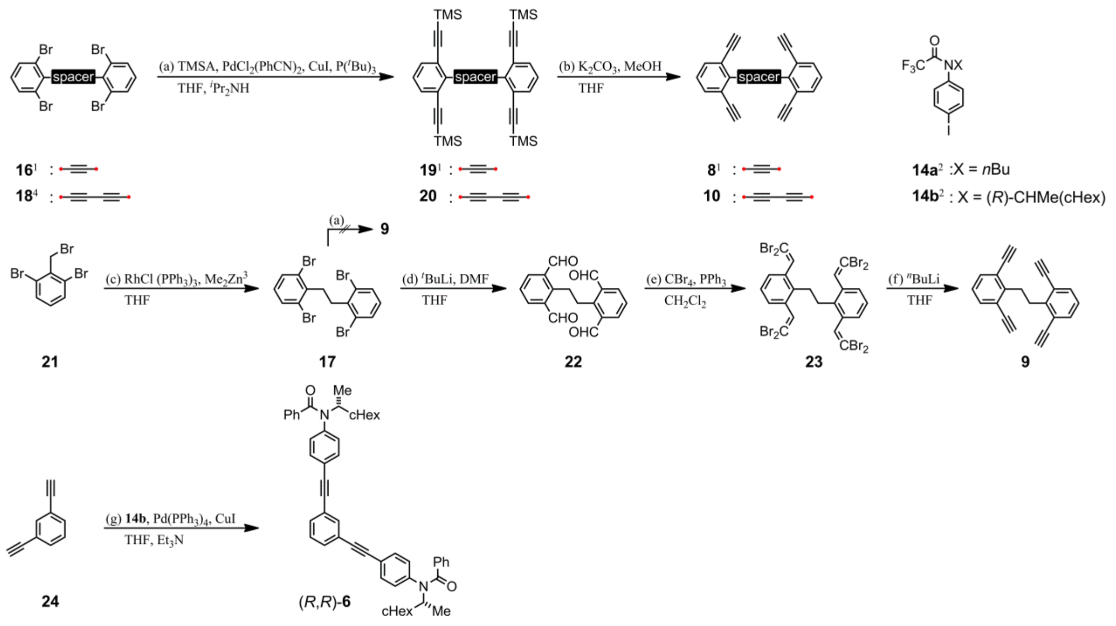

Scheme S1. Preparation of tetraynes **8-10** and single-stranded model (*R*,*R*)-**6**. Yields: (a) 77% for **19**, 88% for **20**; (b) 97% for **10**; (c) 66%; (d) 35%; (e) 90%; (f) 73%; (g) 95%.

Experimental

Preparation of **19**[1]

To a solution of **16**[1] (799 mg, 1.62 mmol), $P(^tBu)_3$ (1.5 mL of 5.0 mmol/10 mL toluene solution), $PdCl_2(PhCN)_2$ (146 mg, 0.381 mmol), and CuI (73 mg, 0.38 mmol) in THF (12 mL) and $^iPr_2NH$ (5 mL) was added TMSA (2.3 mL, 16 mmol) at room temperature under an argon atmosphere, and the mixture was stirred for 2 hours. To the reaction mixture were added aq. HCl and chloroform, and the organic layer was separated, concentrated, and purified by column chromatography on SiO_2 (hexane) to give **19** (700 mg) as a brownish-white solid in 77% yield. The TMS groups were removed with K_2CO_3 in MeOH and THF to give **8**, according to the literature.

Preparation of **11a'** (TFA-protected) [X = *n*Bu]

To a solution of **8**[1] (206 mg, 0.751 mmol) and **14a**[2] (1.34 g, 3.61 mmol) in Et_2NH (26 mL) and CH_3CN (130 mL) were added $Pd(PPh_3)_4$ (60 mg, 0.052 mmol) and CuI (18 mg, 0.095 mmol) at 80 °C under an argon atmosphere, and the mixture was stirred at that temperature for 2 hours. The reaction mixture was diluted with ethyl acetate, and the organic layer was washed with water, concentrated, and then purified by column chromatography on SiO_2 (dichloromethane) to give **11a'** (701 mg) as a yellowish-white amorphous solid in 75% yield. An analytical sample was obtained as a yellowish-white solid through purification by GPC (chloroform, RI detected). **11a'**: mp 79-81 °C; elemental analyses Found: C, 66.78; H, 4.78; N, 4.48%. Calc. for $C_{70}H_{58}N_4O_4F_{12}$·(EtOH): C, 66.87;

H, 4.99; N, 4.33%; IR (KBr) ν_{max}/cm^{-1} 3061, 2960, 2934, 2874, 2212, 1698, 1510, 1209, 1149; ^{1}H NMR δ_H(400 MHz; $CDCl_3$; Me_4Si)/ppm 7.63 (4H, d, J = 7.6 Hz), 7.44-7.40 (8+2H, m), 7.02 (8H, d, J = 8.0 Hz), 3.66 (8H, t, J = 7.2 Hz), 1.47 (8H, quin, J = 7.2 Hz), 1.30 (8H, sext, J = 7.2 Hz), 0.89 (12H, t, J = 7.2 Hz); ^{13}C NMR δ_C(100 MHz; $CDCl_3$)/ppm 156.4 ($\underline{C}$(=O)CF_3), 138.8, 132.8, 131.8, 128.3, 128.0, 128.0, 126.0, 123.7, 116.3 ($\underline{C}F_3$), 94.9, 93.0, 89.3, 51.5, 28.8, 19.8, 13.6; FD-LRMS *m*/*z* 1246.40 (M^+, 100%), 1247.41 ($[M+1]^+$, 79), 1248.41 ($[M+2]^+$, 33), 1249.41 ($[M+3]^+$, 10); FD-HRMS Found: 1246.42712. Calc. for $C_{70}H_{58}N_4O_4F_{12}$: 1246.42664.

Preparation of **1a** [X = *n*Bu]

To an ice-cooled solution of **11a'** (394 mg, 0.316 mmol) in THF (13 mL) were added 60% NaH in oil (628 mg, 15.7 mmol) and MeOH (13 mL), and the mixture was stirred at room temperature for 1 hour, and then diluted with dichloromethane. The organic layer was washed with water, concentrated, and then purified by column chromatography on Al_2O_3 (1:1 dichloromethane/hexane) to give tetraaniline **11a** (147 mg) as a yellow solid in 54% yield, which was immediately subjected to the next reaction.

To a solution of **11a** (147 mg, 0.170 mmol) in toluene (33 mL) and THF (33 mL) containing Et_3N (0.47 mL, 3.4 mmol) was added at 80 °C terephthaloyl chloride in two portions (77 mg, 0.38 mmol and 39 mg, 0.19 mmol) over a period of 4 hours. The reaction mixture was diluted with chloroform and water. The organic layer was separated, concentrated, and purified by column chromatography on SiO_2 (chloroform), followed by GPC (chloroform, RI detected) to give **1a** (157 mg) as a white solid in 82% yield. An analytical sample was suspended in refluxed EtOH, and collected as a white solid through filtration. A single crystal was obtained as colorless needles by recrystallization from chloroform with a diffused vapor of benzene. **1a**: mp >300 °C; elemental analyses Found: C, 82.86; H, 5.95; N, 4.89%. Calc. for $C_{78}H_{66}N_4O_4{\cdot}(H_2O)_{0.5}$: C, 82.73; H, 5.96; N, 4.95%; UV λ_{max}(CH_2Cl_2)/nm 362sh (log ε 4.13), 315 (5.12) and 300sh (5.02); IR (KBr) ν_{max}/cm^{-1} 3058, 2956, 2930, 2870, 2208, 1658, 1510; ^{1}H NMR δ_H(400 MHz; $CDCl_3$; Me_4Si)/ppm 7.62 (4H, d, J = 8.0 Hz), 7.41 (8H, d, J = 8.4 Hz), 7.36 (2H, t, J = 8.0 Hz), 6.97 (8H, s), 6.79 (8H, br s), 4.10-4.00 (4H, m), 3.61-3.54 (4H, m), 1.54-1.45 (8H, m), 1.37-1.27 (8H, m), 0.87 (12H, t, J = 7.2 Hz); ^{13}C NMR δ_C(100 MHz; $CDCl_3$)/ppm 170.0, 142.9, 137.4, 132.7, 132.6, 128.4, 128.1, 127.3, 126.6, 126.2, 121.4, 94.2, 92.9, 89.3, 49.1, 29.7, 20.1, 13.8; FD-LRMS *m*/*z* 1122.64 (M^+, 100%), 1123.64 ($[M+1]^+$, 86), 1124.65 ($[M+2]^+$, 39), 1125.65 ($[M+3]^+$, 13); FD-HRMS Found: 1122.50852. Calc. for $C_{78}H_{66}N_4O_4$: 1122.50840.

Preparation of **11b'** (TFA-protected) [X = (*R*)-CHMe(cHex)]

To a solution of **8** (199 mg, 0.725 mmol) and (*R*)-**14b**[2] (1.50 g, 3.53 mmol) in Et_2NH (26 mL) and CH_3CN (126 mL) were added $Pd(PPh_3)_4$ (57 mg, 0.049 mmol) and CuI (17 mg, 0.089

mmol) at 80 °C under an argon atmosphere, and the mixture was stirred at that temperature for 17 hours. The reaction mixture was diluted with ethyl acetate, and the organic layer was washed with water, concentrated, and then purified by column chromatography on SiO_2 (5:1 chloroform/hexane) to give **11b'** (804 mg) as a yellowish-white solid in 76% yield. An analytical sample was obtained as a yellowish-brown solid through purification by GPC (chloroform, RI detected). **11b'**: mp 110.0-112.0 °C; $[\alpha]_D^{26}$ –59.0 (*c* 1.00 in $CHCl_3$); elemental analyses Found: C, 69.83; H, 5.64; N, 3.70%. Calc. for $C_{86}H_{82}N_4O_4F_{12}\cdot(EtOH)$: C, 70.01; H, 5.88; N, 3.71%; IR (KBr) ν_{max}/cm^{-1} 3061, 2932, 2853, 2213, 1696, 1508, 1208, 1188, 1149; ^1H NMR δ_H(400 MHz; $CDCl_3$; Me_4Si)/ppm 7.63 (4H, d, *J* = 7.6 Hz), 7.42 (2H, t, *J* = 7.6 Hz), 7.41 (8H, d, *J* = 8.0 Hz), 6.99 (8H, d, *J* = 8.0 Hz), 4.34 (4H, dq, *J* = 6.8, 10 Hz), 1.92 (4H, d, *J* = 12 Hz), 1.80-1.60 (16H, m), 1.41-0.84 (24H, m), 1.03 (12H, d, *J* = 6.8 Hz); ^{13}C NMR δ_C(100 MHz; $CDCl_3$)/ppm 156.7 ($\underline{C}$(=O)CF_3), 135.9, 132.3, 132.1, 131.8, 130.3, 129.0, 128.3, 128.1, 126.0, 123.8, 116.4 ($\underline{C}F_3$), 95.0, 93.1, 89.4, 59.8, 40.2, 30.6, 29.7, 26.1, 25.9, 25.7, 16.2; FD-LRMS *m/z* 1462.78 (M^+, 100%), 1463.78 ($[M+1]^+$, 98), 1464.79 ($[M+2]^+$, 51), 1465.79 ($[M+3]^+$, 18); FD-HRMS Found: 1462.61329. Calc. for $C_{86}H_{82}N_4O_4F_{12}$: 1462.61444.

Preparation of (*R*,*R*,*R*,*R*)-**1b** [X = (*R*)-CHMe(cHex)]

To an ice-cooled solution of **11b'** (402 mg, 0.275 mmol) in THF (11 mL) were added 60% NaH in oil (925 mg, 23.1 mmol) and MeOH (11 mL), and the mixture was stirred at room temperature for 1 hour, and then diluted with dichloromethane. The organic layer was washed with water, concentrated, and then purified by column chromatography on Al_2O_3 (1:1 dichloromethane/hexane) to give tetraaniline **11b** (239 mg) as a yellowish-white solid in 81% yield, which was immediately subjected to the next reaction.

To a solution of **11b** (239 mg, 0.221 mmol) in toluene (43 mL) and THF (43 mL) containing Et_3N (0.62 mL, 4.5 mmol) was added at 80 °C terephthaloyl chloride in four portions (100 mg, 0.493 mmol, 101 mg, 0.498 mmol, 295 mg, 1.45 mmol and 297 mg, 1.46 mmol) over a period of 7 hours. The reaction mixture was diluted with chloroform and water. The organic layer was separated, concentrated, and purified by column chromatography on SiO_2 (chloroform), followed by GPC (chloroform, RI detected) to give (*R*,*R*,*R*,*R*)-**1b** (52 mg) as a white solid in 18% yield. An analytical sample was suspended in refluxed EtOH, and collected as a white solid through filtration. (*R*,*R*,*R*,*R*)-**1b**: mp 228-272 °C (dec); $[\alpha]_D^{24}$ –280 (*c* 1.00 in $CHCl_3$); elemental analyses Found: C, 82.85; H, 6.75; N, 4.11%. Calc. for $C_{94}H_{90}N_4O_4\cdot(H_2O)$: C, 83.15; H, 6.83; N, 4.13%; UV $\lambda_{max}(CH_2Cl_2)$/nm 363sh (log ε 4.12), 314 (5.13) and 300sh (5.03); CD $\lambda(CH_2Cl_2)$/nm 363 ($\Delta\varepsilon$ –5.6), 358 (–4.8), 332 (–60.8), 311 (+224.9), 293 (–118.7), 291 (–117.6), 282sh (–161.6) and 273 (–168.2); IR (KBr) ν_{max}/cm^{-1} 3059, 2927, 2851, 2209, 1658, 1509; ^1H NMR δ_H(400 MHz; $CDCl_3$; Me_4Si)/ppm (303K) [two sets] [7.65, 7.63] (4H, [d, *J* = 7.6 Hz]×2), 7.45 (4H, br d), [7.37, 7.36] (2H, [t, *J* = 7.6 Hz]×2), 7.36 (4H, br d), 7.11 (4H, br d), [6.93, 6.92] (8H, s×2), [6.48, 6.39] (4H, br d×2),

[4.57, 4.36] (4H, [dq, J = 6.8, 10 Hz]×2), [2.16, 1.99] (4H, [br d]×2), 1.84-1.48 (16H, m), 1.33-0.88 (24H, m), [1.24, 0.99] (12H, [d, J = 6.8 Hz]×2); ^{13}C NMR δ_C(100 MHz; $CDCl_3$)/ppm (296 K) [two sets] [170.6, 170.3], [141.2, 140.8], [138.0, 137.9], 132.6, 132.3, 131.6, 130.4, 129.7, 128.4, 128.0, 127.1, [126.8, 126.7], [126.2, 126.2], 121.6, [94.2, 94.1], [92.9, 92.9], 89.4, [58.8, 56.9], [42.0, 40.0], [31.2, 30.6], [30.2, 30.0], 26.2, [26.0, 26.0], 25.9, [17.1, 16.5]; FD-LRMS *m*/*z* 1118.63 ([M−2×CHMe(cHex)+2]$^+$, 7%), 1119.62 ([M−2×CHMe(cHex)+3]$^+$, 6), 1228.74 ([M−CHMe(cHex)+1]$^+$, 47), 1229.75 ([M−CHMe(cHex)+2]$^+$, 43), 1230.75 ([M−CHMe(cHex)+3]$^+$, 22), 1338.87 (M$^+$, 93), 1339.87 ([M+1]$^+$, 100), 1340.87 ([M+2]$^+$, 56), 1341.88 ([M+3]$^+$, 22), 1342.89 ([M+4]$^+$, 6); FD-HRMS Found: 1338.69741. Calc. for $C_{94}H_{90}N_4O_4$: 1338.69620.

Preparation of **17**

To a solution of **21** (10.9 g, 33.2 mmol) and $RhCl(PPh_3)_3$ (624 mg, 0.674 mmol) in THF (166 mL) was added a 1M solution of Me_2Zn in heptane (34 mL) at room temperature under an argon atmosphere,[3] the mixture was refluxed for 17 h. The reaction was quenched with aq. HCl, and extracted with ethyl acetate. The organic layer was separated, concentrated, and purified by column chromatography on SiO_2 (dichloromethane), followed by GPC (chloroform, RI detected) to give **17** (5.47 g) as a white solid in 66% yield. **17**: mp 159.0-160.0 °C; elemental analyses Found: C, 33.63; H, 2.20%. Calc. for $C_{14}H_{10}Br_4$: C, 33.78; H, 2.02%; IR (KBr) ν_{max}/cm^{-1} 2946, 1551, 1447, 1426, 1153, 1107, 764, 720, 707; ^{1}H NMR δ_H(400 MHz; $CDCl_3$; Me_4Si)/ppm 7.47 (4H, d, J = 8.0 Hz), 6.91 (2H, t, J = 8.0 Hz), 3.37 (4H, s); ^{13}C NMR δ_C(100 MHz; $CDCl_3$)/ppm 139.2, 132.3, 128.7, 126.0, 34.9; FD-LRMS *m*/*z* 493.79 (M$^+$, 18%), 495.79 ([M+2]$^+$, 70), 496.79 ([M+3]$^+$, 11), 497.78 ([M+4]$^+$, 100), 498.79 ([M+5]$^+$, 16), 499.78 ([M+6]$^+$, 65), 500.79 ([M+7]$^+$, 10), 501.78 ([M+8]$^+$, 17).

Preparation of **22**

To an acetone-dry ice-cooled solution of **17** (200 mg, 0.402 mmol) in THF (13 mL) was added a 1.6M solution of ^{t}BuLi in pentane (4 mL), and the mixture was stirred at that temperature for 1 hour. To the mixture was added DMF (0.5 mL, 6 mmol), and the mixture was stirred at that temperature for 5 hours, followed by stirring at room temperature for 16 hours. The reaction mixture was quenched with aq. NH_4Cl, and extracted with dichloromethane. The organic layer was separated, concentrated, and purified by column chromatography on SiO_2 (dichloromethane) to give **22** (41 mg) as a white solid in 35% yield. An analytical sample was suspended in dichloromethane and hexane, and collected as a white solid through filtration. **22**: mp 206.5-208.0 °C; IR (KBr) ν_{max}/cm^{-1} 3086, 2914, 2872, 2812, 2777, 1686, 1577; ^{1}H NMR δ_H(400 MHz; $CDCl_3$; Me_4Si)/ppm 10.25 (4H, s), 8.06 (4H, d, J = 7.6 Hz), 7.61 (2H, t, J = 7.6 Hz), 3.89 (4H, s); ^{13}C NMR δ_C(100 MHz; $CDCl_3$)/ppm 191.5, 144.4, 137.2, 135.5, 127.6, 27.6; FD-LRMS *m*/*z* 276.05 ([M−(H_2O)]$^+$, 100%), 294.06 (M$^+$, 83); FD-HRMS Found: 294.09089. Calc. for $C_{18}H_{14}O_4$: 294.08921.

Preparation of **12a'** (TFA-protected) [X = *n*Bu]

To an ice-cooled solution of **22** (261 mg, 0.887 mmol) and CBr_4 (3.52 g, 10.6 mmol) in CH_2Cl_2 (110 mL) was added PPh_3 (5.57 g, 21.3 mmol), the mixture was stirred at room temperature for 15 min. After removal of a solid by filtration, the filtrate was concentrated and purified by column chromatography on SiO_2 (dichloromethane) to give **23** (736 mg) as a white amorphous solid in 90% yield, which was immediately subjected to the next reaction.

To an acetone-dry ice-cooled solution of **23** (1.65 g, 1.80 mmol) in THF (405 mL) was added a 1.6M solution of ^{n}BuLi in hexane (9 mL), and the mixture was stirred at that temperature for 10 min. The reaction mixture was quenched with aq. NH_4Cl, and extracted with ethyl acetate. The organic layer was separated, washed with brine, concentrated, and purified by column chromatography on SiO_2 (1:4 dichloromethane/hexane) to give **9** (365 mg) as a white solid in 73% yield, which was subjected to the next reactions without further purification.

To a solution of **9** (158 mg, 0.568 mmol) and **14a** (1.02 g, 2.74 mmol) in Et_3N (23 mL) and THF (23 mL) were added $Pd(PPh_3)_4$ (182 mg, 0.158 mmol) and CuI (59 mg, 0.31 mmol) at 60 °C under an argon atmosphere, and the mixture was stirred at that temperature for 14 hours. The reaction mixture was diluted with ethyl acetate, and the organic layer was washed with water, concentrated, and then purified by column chromatography on SiO_2 (1:1 dichloromethane/hexane) to give **12a'** (274 mg) as a yellowish-white solid in 39% yield. An analytical sample was obtained as a yellowish-white solid through purification by GPC (chloroform, RI detected). **12a'**: mp 169.0-171.0 °C; elemental analyses Found: C, 66.98; H, 4.97; N, 4.48%. Calc. for $C_{70}H_{62}N_4O_4F_{12}$: C, 67.19; H, 4.99; N, 4.48%; IR (KBr) ν_{max}/cm^{-1} 3064, 2961, 2934, 2874, 2210, 1698, 1510, 1210, 1150; ^{1}H NMR δ_H(400 MHz; $CDCl_3$; Me_4Si)/ppm 7.53 (8H, d, J = 8.4 Hz), 7.21 (4H, d, J = 8.0 Hz), 7.19 (8H, d, J = 8.4 Hz), 7.00 (2H, t, J = 8.0 Hz), 3.76 (8H, t, J = 7.6 Hz), 3.74 (4H, s), 1.57 (8H, quin, J = 7.6 Hz), 1.36 (8H, sext, J = 7.6 Hz), 0.93 (12H, t, J = 7.6 Hz); ^{13}C NMR δ_C(100 MHz; $CDCl_3$)/ppm 156.4 ($\underline{C}$(=O)CF_3), 144.9, 138.7, 132.6, 132.4, 128.3, 126.0, 124.3, 123.5, 116.4 ($\underline{C}F_3$), 91.5, 89.6, 51.6, 32.7, 28.9, 19.8, 13.7; FD-LRMS m/z 1250.53 (M^+, 100%), 1251.53 ($[M+1]^+$, 79), 1252.53 ($[M+2]^+$, 33), 1253.54 ($[M+3]^+$, 9).

Preparation of **2a** [X = *n*Bu]

To an ice-cooled solution of **12a'** (178 mg, 0.142 mmol) in THF (5.6 mL) were added 60% NaH in oil (248 mg, 6.20 mmol) and MeOH (5.6 mL), and the mixture was stirred at room temperature for 20 min, and then diluted with dichloromethane. The organic layer was washed with water, concentrated, and then purified by column chromatography on Al_2O_3 (hexane-dichloromethane) to give tetraaniline **12a** (118 mg) as a yellow solid in 96% yield, which was immediately subjected to the next reaction.

To a solution of **12a** (118 mg, 0.136 mmol) in toluene (26 mL) and THF (26 mL) containing Et_3N (0.38 mL, 2.7 mmol) was added at 80 °C terephthaloyl chloride (62 mg, 0.31 mmol), and the mixture was stirred at that temperature for 75 min. The reaction mixture was diluted with chloroform and water. The organic layer was separated, concentrated, and purified by column chromatography on SiO_2 (1:1 ethyl acetate/chloroform), followed by GPC (chloroform, RI detected) to give **2a** (96 mg) as a white solid in 63% yield. An analytical sample was suspended in refluxed EtOH, and collected as a white solid through filtration. **2a**: mp >300 °C; elemental analyses Found: C, 82.93; H, 6.26; N, 4.95%. Calc. for $C_{78}H_{70}N_4O_4$: C, 83.10; H, 6.26; N, 4.97%; UV $\lambda_{max}(CH_2Cl_2)$/nm 315sh (log ε 4.98) and 299 (5.08); IR (KBr) ν_{max}/cm^{-1} 3061, 2957, 2930, 2870, 2208, 1656, 1509; ^1H NMR δ_H(400 MHz; $CDCl_3$; Me_4Si)/ppm 7.55 (4H, d, J = 7.6 Hz), 7.39 (8H, d, J = 8.0 Hz), 7.22 (2H, t, J = 7.6 Hz), 7.01 (8H, s), 6.81 (8H, d, J = 8.0 Hz), 3.85 (8H, br t), 3.78 (4H, br s), 1.53 (8H, quin, J = 7.6 Hz), 1.33 (8H, sext, J = 7.6 Hz), 0.88 (12H, t, J = 7.6 Hz); ^{13}C NMR δ_C(100 MHz; $CDCl_3$)/ppm 169.8, 144.3, 142.8, 137.3, 133.5, 132.2, 128.2, 127.4, 126.2, 123.6, 121.6, 92.5, 89.6, 49.3, 32.4, 29.7, 20.1, 13.8; FD-LRMS m/z 1126.58 (M^+, 100%), 1127.59 ($[M+1]^+$, 91), 1128.59 ($[M+2]^+$, 42), 1129.60 ($[M+3]^+$, 14).

Preparation of **12b'** (TFA-protected) [X = (*R*)-CHMe(cHex)]

To a solution of **9** (365 mg, 1.31 mmol) and (*R*)-**14b** (2.68 g, 6.30 mmol) in Et_3N (52 mL) and THF (52 mL) were added $Pd(PPh_3)_4$ (422 mg, 0.365 mmol) and CuI (138 mg, 0.724 mmol) at 60 °C under an argon atmosphere, and the mixture was stirred at that temperature for 9 hours. The reaction mixture was diluted with ethyl acetate, and the organic layer was washed with water, concentrated, and then purified by column chromatography on SiO_2 (1:1 dichloromethane/hexane-dichloromethane) to give **12b'** (1.57 g) as a yellowish-white amorphous solid in 82% yield. An analytical sample was obtained as a yellowish-white solid through purification by GPC (chloroform, RI detected). **12b'**: mp 112.0-114.0 °C; $[\alpha]_D^{26}$ –37.4 (c 1.00 in $CHCl_3$); elemental analyses Found: C, 69.40; H, 5.82; N, 3.66%. Calc. for $C_{86}H_{86}N_4O_4F_{12}\cdot(H_2O)$: C, 69.53; H, 5.97; N, 3.77%; IR (KBr) ν_{max}/cm^{-1} 3063, 2931, 2853, 2209, 1695, 1508, 1208, 1188, 1148; ^1H NMR δ_H(400 MHz; $CDCl_3$; Me_4Si)/ppm 7.52 (8H, d, J = 7.2 Hz), 7.21 (4H, d, J = 7.6 Hz), 7.16 (8H, br d), 7.01 (2H, t, J = 7.6 Hz), 4.44 (4H, dq, J = 6.8, 10 Hz), 3.76 (4H, s), 1.99 (4H, br d), 1.87-1.57 (16H, m), 1.52-1.36 (4H, m), 1.32-0.91 (20H, m), 1.13 (12H, d, J = 6.8 Hz); ^{13}C NMR δ_C(100 MHz; $CDCl_3$)/ppm 156.8 ($\underline{C}(=O)CF_3$), 144.9, 135.8, 132.6, 131.9, 131.7, 130.6, 129.3, 126.0, 124.4, 123.5, 116.5 ($\underline{C}F_3$), 91.5, 89.7, 59.8, 40.3, 32.7, 30.7, 29.7, 26.1, 25.9, 25.8, 16.4; FD-LRMS m/z 1466.84 (M^+, 100%), 1467.85 ($[M+1]^+$, 97), 1468.84 ($[M+2]^+$, 53) , 1469.85 ($[M+3]^+$, 19); FD-HRMS Found: 1466.64672. Calc. for $C_{86}H_{86}N_4O_4F_{12}$: 1466.64574.

Preparation of (*R*,*R*,*R*,*R*)-**2b** [X = (*R*)-CHMe(cHex)]

To an ice-cooled solution of **12b'** (401 mg, 0.273 mmol) in THF (11 mL) were added 60% NaH in oil (440 mg, 11.0 mmol) and MeOH (11 mL), and the mixture was stirred at room temperature for 40 min, and then diluted with dichloromethane. The organic layer was washed with water, concentrated, and then purified by column chromatography on Al_2O_3 (hexane-1:5 ethyl acetate/hexane) to give tetraaniline **12b** (209 mg) as a white amorphous solid in 71% yield, which was immediately subjected to the next reaction.

To a solution of **12b** (209 mg, 0.193 mmol) in toluene (37 mL) and THF (37 mL) containing Et_3N (0.55 mL, 4.0 mmol) was added at 80 °C terephthaloyl chloride (418 mg, 2.06 mmol), and the mixture was stirred at that temperature for 3.5 hours. The reaction mixture was diluted with chloroform and water. The organic layer was separated, concentrated, and purified by column chromatography on Al_2O_3 (1:5 ethyl acetate/hexane-ethyl acetate), followed by GPC (chloroform, RI detected) to give (*R*,*R*,*R*,*R*)-**2b** (102 mg) as a white solid in 39% yield. An analytical sample was suspended in refluxed EtOH, and collected as a white solid through filtration. (*R*,*R*,*R*,*R*)-**2b**: mp >300 °C; $[\alpha]_D^{26}$ –393 (*c* 1.00 in $CHCl_3$); elemental analyses Found: C, 82.83; H, 6.84; N, 4.10%. Calc. for $C_{94}H_{94}N_4O_4 \cdot (H_2O)$: C, 82.91; H, 7.11; N, 4.11%; UV $\lambda_{max}(CH_2Cl_2)$/nm 317sh (log ε 5.02) and 299 (5.11); CD $\lambda(CH_2Cl_2)$/nm 321 ($\Delta\varepsilon$ –111.9), 314 (–58.4), 312 (–57.6), 295 (+25.5), 294 (+25.2), 289 (+29.7), 275 (+4.1), 274 (+4.2) and 258 (–61.9); IR (KBr) ν_{max}/cm^{-1} 3063, 2927, 2851, 2203, 1656, 1508; ^1H NMR δ_H(400 MHz; $CDCl_3$; Me_4Si)/ppm (303 K) 7.56 (4H, d, *J* = 8.0 Hz), 7.39 (8H, d, *J* = 8.8 Hz), 7.24 (2H, t, *J* = 8.0 Hz), 6.95 (8H, s), 6.77 (8H, br s), 4.55-4.46 (4H, m), 2.10 (4H, br d), 1.84-1.48 (20H, br m), 1.31-0.91 (32H, br m); ^{13}C NMR δ_C(100 MHz; $CDCl_3$)/ppm 170.5, 144.6, 140.8, 138.0, 133.7, 131.9, 129.9, 127.1, 126.2, 123.5, 121.7, 92.8, 89.9, 57.6, 41.4, 32.6, 30.8, 30.2, 26.2, 26.0, 26.0, 16.8; FD-LRMS *m*/*z* 1342.90 (M^+, 91%), 1343.90 ($[M+1]^+$, 100), 1344.91 ($[M+2]^+$, 58), 1345.91 ($[M+3]^+$, 22), 1346.91 ($[M+4]^+$, 7); FD-HRMS Found: 1342.72602. Calc. for $C_{94}H_{94}N_4O_4$: 1342.72750.

Preparation of **20**

To a solution of **18**[4] (790 mg, 1.53 mmol), $P(^tBu)_3$ (1 mL of 5.0 mmol/10 mL toluene solution), $PdCl_2(PhCN)_2$ (138 mg, 0.360 mmol), and CuI (69 mg, 0.36 mmol) in THF (12 mL) and $^iPr_2NH$ (4 mL) was added TMSA (3.3 mL, 23 mmol) at room temperature under an argon atmosphere, and the mixture was stirred for 3 hours, followed by addition of TMSA (1.1 mL, 7.8 mmol) and stirring for additional 1 hour. To the reaction mixture were added aq. HCl and chloroform, and the organic layer was separated, concentrated, and purified by column chromatography on SiO_2 (hexane-dichloromethane) to give **20** (787 mg) as a brownish-white solid in 88% yield. An analytical sample was obtained as a white solid through purification by GPC (chloroform, RI detected). **20**: mp 148.0-149.0 °C; elemental analyses Found: C, 73.57; H, 7.17%. Calc. for $C_{36}H_{42}Si_4$: C, 73.65; H, 7.21%; IR (KBr) ν_{max}/cm^{-1} 3065, 2959, 2898, 2155, 1556, 1455,

1250, 979, 841; ^{1}H NMR δ_H(400 MHz; $CDCl_3$; Me_4Si)/ppm 7.40 (4H, d, J = 8.0 Hz), 7.20 (2H, t, J = 8.0 Hz), 0.28 (36H, s); ^{13}C NMR δ_C(100 MHz; $CDCl_3$)/ppm 131.6, 128.0, 128.0, 127.1, 102.5, 100.1, 81.9, 80.6, 0.0; FD-LRMS *m/z* 586.24 (M^+, 100%), 587.24 ($[M+1]^+$, 59), 588.24 ($[M+2]^+$, 30), 589.24 ($[M+3]^+$, 10).

Preparation of **13a'** (TFA-protected) [X = *n*Bu]

A mixture of **20** (837 mg, 1.43 mmol), K_2CO_3 (789 mg, 5.71 mmol), MeOH (29 mL), and THF (29 mL) was stirred at room temperature for 1.5 hour, and diluted with ethyl acetate and water. The organic layer was separated, concentrated, and purified by column chromatography on Al_2O_3 (hexane-1:1 dicchloromethane/hexane) to give **10** (412 mg) as a brownish-white solid in 97%, which was subjected to the next reactions without further purification.

To a solution of **10** (220 mg, 0.738 mmol) and **14a** (1.24 g, 3.34 mmol) in Et_2NH (25 mL) and CH_3CN (120 mL) were added $Pd(PPh_3)_4$ (54 mg, 0.047 mmol) and CuI (16 mg, 0.084 mmol) at 80 °C under an argon atmosphere, and the mixture was stirred at that temperature for 8 hours. The reaction mixture was diluted with ethyl acetate, and the organic layer was washed with water, concentrated, and then purified by column chromatography on SiO_2 (1:1 dichloromethane/hexane-dichloromethane) to give **13a'** (714 mg) as an orange amorphous solid in 75% yield. An analytical sample was obtained as an orange solid through purification by GPC (chloroform, RI detected). **13a'**: mp 69-71 °C; elemental analyses Found: C, 67.40; H, 4.68; N, 4.32%. Calc. for $C_{72}H_{58}N_4O_4F_{12}\cdot(EtOH)$: C, 67.47; H, 4.90; N, 4.25%; IR (KBr) ν_{max}/cm^{-1} 3059, 2961, 2936, 2874, 2214, 1698, 1510, 1209, 1150; ^{1}H NMR δ_H(400 MHz; $CDCl_3$; Me_4Si)/ppm 7.62 (8H, d, J = 8.4 Hz), 7.58 (4H, d, J = 7.6 Hz), 7.42 (2H, t, J = 7.6 Hz), 7.10 (8H, d, J = 8.4 Hz), 3.63 (8H, t, J = 7.2 Hz), 1.46 (8H, quin, J = 7.2 Hz), 1.27 (8H, sext, J = 7.2 Hz), 0.87 (12H, t, J = 7.2 Hz); ^{13}C NMR δ_C(100 MHz; $CDCl_3$)/ppm 156.4 ($\underline{C}(=O)CF_3$), 139.2, 132.8, 131.4, 129.0, 128.3, 127.3, 127.1, 123.4, 116.2 ($\underline{C}F_3$), 93.6, 88.9, 82.0, 81.9, 51.5, 28.8, 19.7, 13.6; FD-LRMS *m/z* 1270.56 (M^+, 100%), 1271.56 ($[M+1]^+$, 85), 1272.56 ($[M+2]^+$, 39), 1273.56 ($[M+3]^+$, 12); FD-HRMS Found: 1270.42487. Calc. for $C_{72}H_{58}N_4O_4F_{12}$: 1270.42664.

Preparation of **3a** [X = *n*Bu]

To an ice-cooled solution of **13a'** (355 mg, 0.276 mmol) in THF (11 mL) were added 60% NaH in oil (883 mg, 22.1 mmol) and MeOH (11 mL), and the mixture was stirred at room temperature for 35 min, and then diluted with dichloromethane. The organic layer was washed with water, concentrated, and then purified by column chromatography on Al_2O_3 (1:1 dichloromethane/hexane) to give tetraaniline **13a** (239 mg) as a yellowish-white amorphous solid in 98% yield, which was immediately subjected to the next reaction.

To a solution of **13a** (239 mg, 0.269 mmol) in toluene (52 mL) and THF (52 mL)

containing Et_3N (0.75 mL, 5.4 mmol) was added at 80 °C terephthaloyl chloride in two portions (120 mg, 0.591 mmol and 62 mg, 0.31 mmol) over a period of 2.5 hours. The reaction mixture was diluted with chloroform and water. The organic layer was separated, concentrated, and purified by column chromatography on SiO_2 (chloroform-1:1 ethyl acetate chloroform), followed by GPC (chloroform, RI detected) to give **3a** (78 mg) as a white solid in 25% yield. An analytical sample was suspended in refluxed EtOH, and collected as a white solid through filtration. **3a**: mp >300 °C; elemental analyses Found: C, 82.51; H, 5.88; N, 4.76%. Calc. for $C_{80}H_{66}N_4O_4$·(EtOH): C, 82.52; H, 6.08; N, 4.69%; UV λ_{max}(CH_2Cl_2)/nm 375 (log ε 4.37), 357sh (4.46), 325 (5.08), 310sh (5.00) and 280sh (4.85); IR (KBr) ν_{max}/cm^{-1} 3055, 2956, 2930, 2870, 2211, 1657, 1510; ^{1}H NMR δ_H(400 MHz; $CDCl_3$; Me_4Si)/ppm 7.55 (4H, d, J = 8.0 Hz), 7.41 (8H, d, J = 8.8 Hz), 7.37 (2H, t, J = 8.0 Hz), 7.00 (8H, s), 6.83 (8H, br d), 4.16 (4H, br s), 3.57 (4H, br s), 1.51 (8H, br s), 1.31 (8H, sext, J = 7.6 Hz), 0.86 (12H, t, J = 7.6 Hz); ^{13}C NMR δ_C(100 MHz; $CDCl_3$)/ppm 169.4, 143.5, 137.2, 132.4, 131.6, 128.7, 128.2, 127.7, 127.2, 125.9, 121.1, 93.6, 88.2, 81.1, 80.7, 49.4, 29.7, 20.1, 13.8; FD-LRMS *m*/*z* 1146.65 (M^+, 100%), 1147.65 ($[M+1]^+$, 91), 1148.65 ($[M+2]^+$, 42), 1149.66 ($[M+3]^+$, 14); FD-HRMS Found: 1146.50630. Calc. for $C_{80}H_{66}N_4O_4$: 1146.50840.

Preparation of **13b'** (TFA-protected) [X = (*R*)-CHMe(cHex)]

To a solution of **10** (192 mg, 0.644 mmol) and (*R*)-**14b** (1.41 g, 3.32 mmol) in Et_2NH (25 mL) and CH_3CN (120 mL) were added $Pd(PPh_3)_4$ (54 mg, 0.047 mmol) and CuI (16 mg, 0.084 mmol) at 80 °C under an argon atmosphere, and the mixture was stirred at that temperature for 8 hours. The reaction mixture was diluted with ethyl acetate, and the organic layer was washed with water, concentrated, and then purified by column chromatography on SiO_2 (1:1 dichloromethane/hexane-dichloromethane) to give **13b'** (834 mg) as a brownish-white solid in 87% yield. An analytical sample was obtained as a white solid through purification by GPC (chloroform, RI detected). **13b'**: mp 113.0-115.0 °C; $[\alpha]_D^{25}$ –145 (*c* 1.00 in $CHCl_3$); elemental analyses Found: C, 70.37; H, 5.54; N, 3.69%. Calc. for $C_{88}H_{82}N_4O_4F_{12}$·(H_2O): C, 70.20; H, 5.62; N, 3.72%; IR (KBr) ν_{max}/cm^{-1} 3061, 2931, 2853, 2211, 1698, 1509, 1208, 1188, 1150; ^{1}H NMR δ_H(400 MHz; $CDCl_3$; Me_4Si)/ppm 7.64 (4H, br d), 7.56 (4H, br d), 7.56 (4H, d, J = 7.6 Hz), 7.42 (2H, t, J = 7.6 Hz), 7.07 (8H, br d), 4.33 (4H, dq, J = 6.8, 10 Hz), 1.90 (4H, br d), 1.82-1.51 (16H, br m), 1.42-0.88 (24H, m), 0.98 (12H, d, J = 6.8 Hz); ^{13}C NMR δ_C(100 MHz; $CDCl_3$)/ppm 156.7 ($\underline{C}$(=O)CF_3), 136.3, 132.5, 132.0, 131.3, 130.8, 129.3, 129.0, 127.4, 127.1, 123.6, 116.3 ($\underline{C}F_3$), 93.7, 89.1, 82.1, 82.0, 59.8, 40.2, 30.5, 29.7, 26.1, 25.9, 25.7, 16.2; FD-LRMS *m*/*z* 1486.75 (M^+, 100%), 1487.75 ($[M+1]^+$, 99), 1488.75 ($[M+2]^+$, 51), 1489.76 ($[M+3]^+$, 19), 1490.76 ($[M+4]^+$, 6); FD-HRMS Found: 1486.61481. Calc. for $C_{88}H_{82}N_4O_4F_{12}$: 1486.61444.

Preparation of (*R*,*R*,*R*,*R*)-**3b** [X = (*R*)-CHMe(cHex)]

To an ice-cooled solution of **13b'** (428 mg, 0.288 mmol) in THF (12 mL) were added 60% NaH in oil (923 mg, 23.1 mmol) and MeOH (12 mL), and the mixture was stirred at room temperature for 40 min, and then diluted with dichloromethane. The organic layer was washed with water, concentrated, and then purified by column chromatography on Al_2O_3 (hexane-2:3 dichloromethane/hexane-dichloromethane) to give tetraaniline **13b** (293 mg) as a yellowish-white solid in 92% yield, which was immediately subjected to the next reaction.

To a solution of **13b** (293 mg, 0.265 mmol) in toluene (51 mL) and THF (51 mL) containing Et_3N (0.74 mL, 5.3 mmol) was added at 80 °C terephthaloyl chloride in three portions (119 mg, 0.586 mmol, 118 mg, 0.581 mmol and 121 mg, 0.596 mmol) over a period of 4 hours, followed by stirring at that temperature for additional 9 hours. The reaction mixture was diluted with chloroform and water. The organic layer was separated, concentrated, and purified by column chromatography on SiO_2 (1:9 ethyl acetate/dichloromethane), followed by GPC (chloroform, RI detected) to give (*R*,*R*,*R*,*R*)-**3b** (136 mg) as a white solid in 38% yield. An analytical sample was suspended in refluxed EtOH, and collected as a white solid through filtration. (*R*,*R*,*R*,*R*)-**3b**: mp >300 °C; $[\alpha]_D^{25}$ –434 (*c* 1.00 in $CHCl_3$); elemental analyses Found: C, 83.14; H, 6.57; N, 4.02%. Calc. for $C_{96}H_{90}N_4O_4{\cdot}(H_2O)$: C, 83.44; H, 6.71; N, 4.05%; UV $\lambda_{max}(CH_2Cl_2)$/nm 375 (log ε 4.36), 357sh (4.45), 326 (5.08), 310 (5.00) and 280sh (4.86); CD $\lambda(CH_2Cl_2)$/nm 378 ($\Delta\varepsilon$ +35.5), 335 (–80.1), 323 (+170.3), 301 (–77.9), 296 (–67.1), 284sh (–99.7) and 271 (–153.8); IR (KBr) ν_{max}/cm^{-1} 3051, 2928, 2852, 2209, 1656, 1509; ^1H NMR δ_H(400 MHz; $CDCl_3$)/ppm (323 K) 7.55 (4H, d, *J* = 8.0 Hz), 7.40 (8H, br d), 7.37 (2H, t, *J* = 8.0 Hz), 6.97 (8H, s), 6.80 (8H, br s), 4.52 (4H, br s), 2.10 (4H, br d), 1.84-1.62 (16H, br m), 1.32-0.93 (36H, br m); ^{13}C NMR δ_C(100 MHz; $CDCl_3$)/ppm (296 K) [two sets] [169.7, 169.6], 141.8, 137.6, 132.0, 131.5,128.7,127.5,127.2, 126.1, 121.2, 93.6, 88.2, 81.1, 80.9, 57.5, 42.5, 30.4, 26.2, 26.0, 26.0, 16.6; FD-LRMS *m*/*z* 1252.74 ($[M–CHMe(cHex)+1]^+$, 27%), 1253.75 ($[M–CHMe(cHex)+2]^+$, 26), 1362.87 (M^+, 90), 1363.87 ($[M+1]^+$, 100), 1364.87 ($[M+2]^+$, 55), 1365.87 ($[M+3]^+$, 23), 1366.88 ($[M+4]^+$, 8); FD-HRMS Found: 1362.69500. Calc. for $C_{96}H_{90}N_4O_4$: 1362.69620.

Preparation of (*R*,*R*)-**6**

To a solution of **24** (0.32 mL, 2.4 mmol) and (*R*)-**14b** (2.50 g, 5.78 mmol) in Et_3N (48 mL) and THF (48 mL) were added $Pd(PPh_3)_4$ (387 mg, 0.335 mmol) and CuI (128 mg, 0.672 mmol) at 62 °C under an argon atmosphere, and the mixture was stirred at that temperature for 1.5 hours. The reaction mixture was diluted with ethyl acetate, and the organic layer was washed with water, concentrated, and then purified by column chromatography on SiO_2 (1:2 dichloromethane/hexane) to give (*R*,*R*)-**6** (1.69 g) as a brownish-white amorphous solid in 95% yield. An analytical sample was obtained as a white amorphous solid through purification by GPC (chloroform, RI detected). (*R*,*R*)-**6**: mp 107.0-109.5 °C; $[\alpha]_D^{26}$ –123 (*c* 1.00 in $CHCl_3$); elemental analyses Found: C, 84.40; H, 7.28;

N, 3.69%. Calc. for $C_{52}H_{52}N_2O_2$: C, 84.75; H, 7.11; N, 3.80%; UV $\lambda_{max}(CH_2Cl_2)$/nm 309 (log ε 4.75) and 298 (4.75); CD $\lambda(CH_2Cl_2)$/nm 310 ($\Delta\varepsilon$ −6.4), 308 (−6.1), 294 (−8.1), 278 (−5.9), 276 (−6.3) and 252 (+1.3); IR (KBr) ν_{max}/cm^{-1} 3058, 2928, 2850, 2211, 1648, 1509; ^{1}H NMR δ_H(400 MHz; $CDCl_3$; Me_4Si)/ppm 7.61 (1H, br t), 7.45-7.42 (2H, m), 7.35 (4H, d, J = 8.4 Hz), 7.30 (1H, dd, J = 7.6, 8.4 Hz), 7.30-7.23 (4H, m), 7.22-7.10 (6H, m), 6.99 (4H, d, J = 8.4 Hz), 4.48 (2H, br s), 2.16 (2H, br d), 1.88-1.58 (10H, br m), 1.36-0.93 (10H, br m), 1.21 (6H, d, J = 6.8 Hz); ^{13}C NMR δ_C(100 MHz; $CDCl_3$)/ppm 170.8, 142.1, 137.1, 134.4, 132.0, 131.4, 129.4, 129.2, 128.5, 128.3, 127.7, 123.3, 121.3, 89.3, 89.1, 59.3, 41.5, 30.8, 30.4, 26.3, 26.1, 26.0, 16.8; FD-LRMS *m*/*z* 736.50 (M^+, 100%), 737.51 ($[M+1]^+$, 63), 738.51 ($[M+2]^+$, 19); FD-HRMS Found: 736.40464. Calc. for $C_{52}H_{52}N_2O_2$: 736.40288.

^{1}H and ^{13}C NMR spectra of new compounds

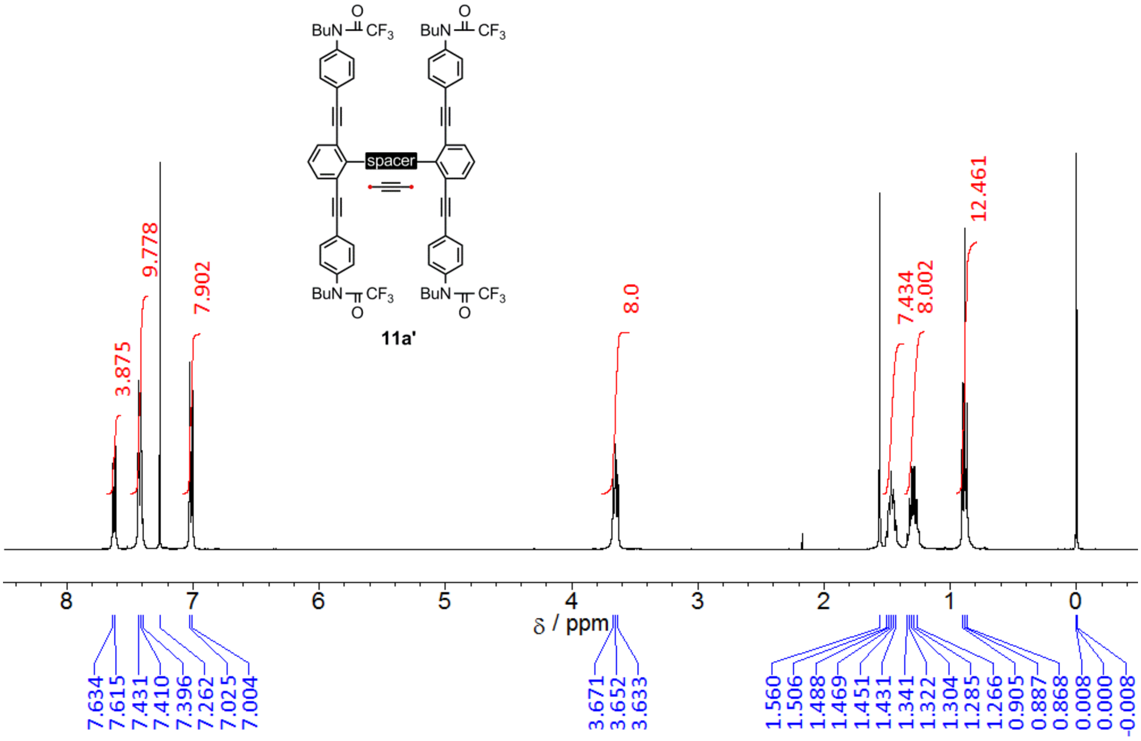

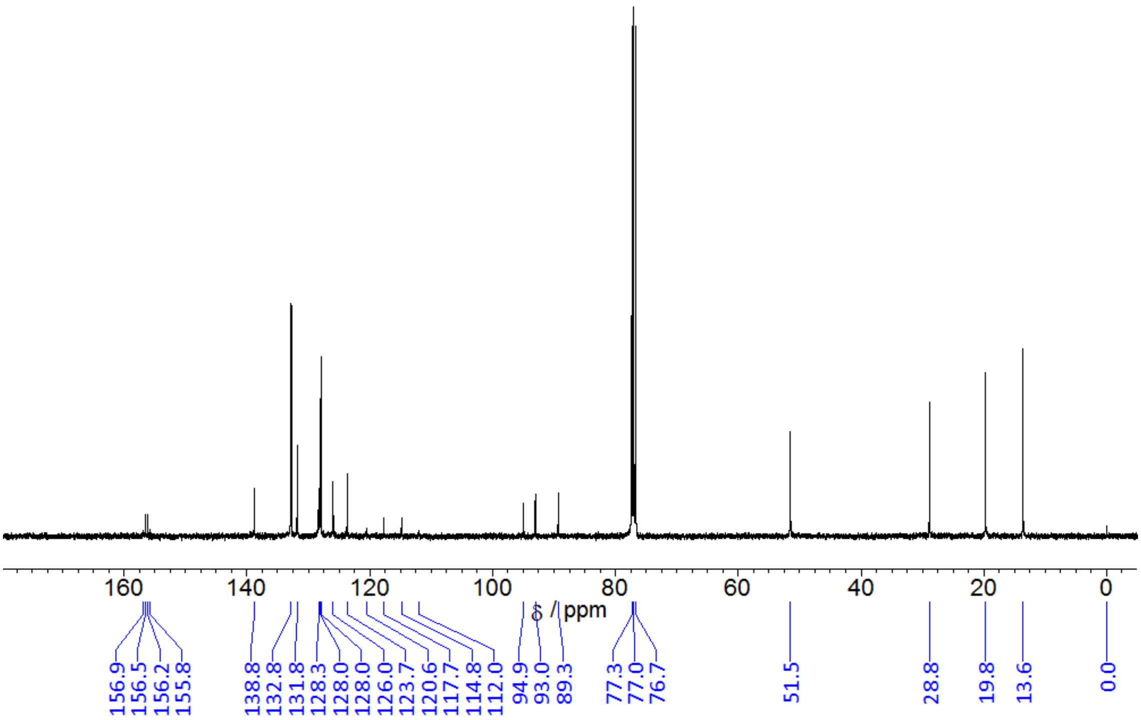

^{1}H (400 MHz, upper) and ^{13}C (100 MHz, lower) NMR spectra of **11a'**, measured in $CDCl_3$ at room temperature.

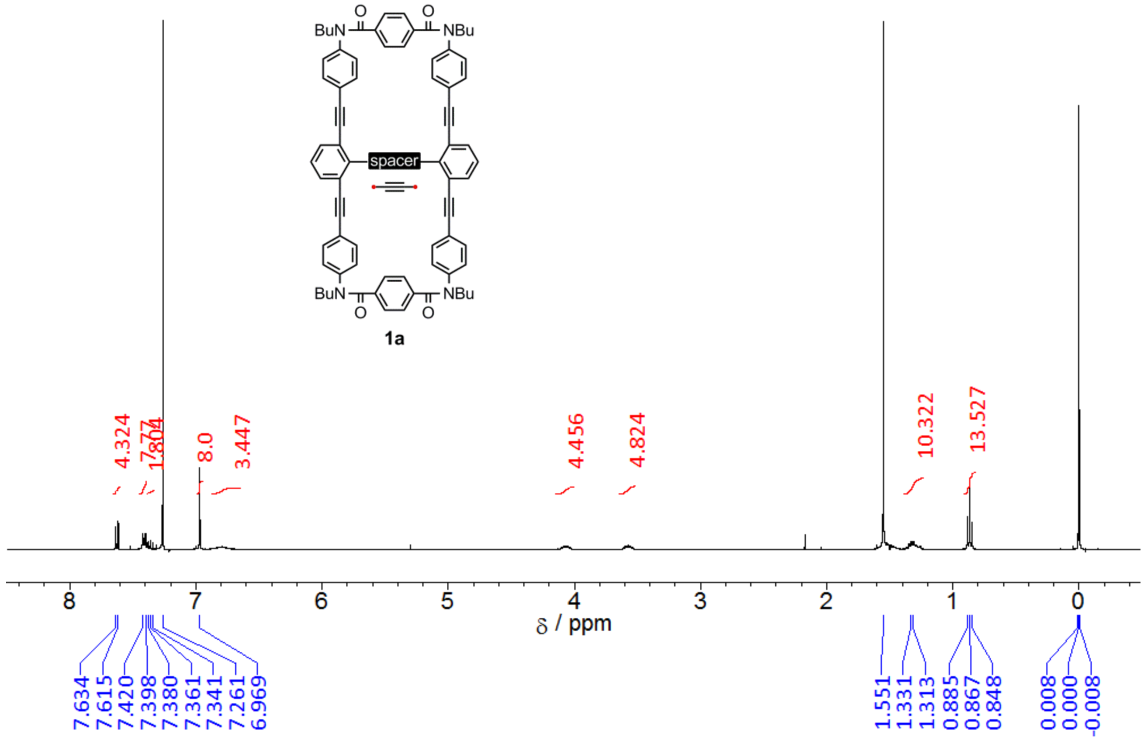

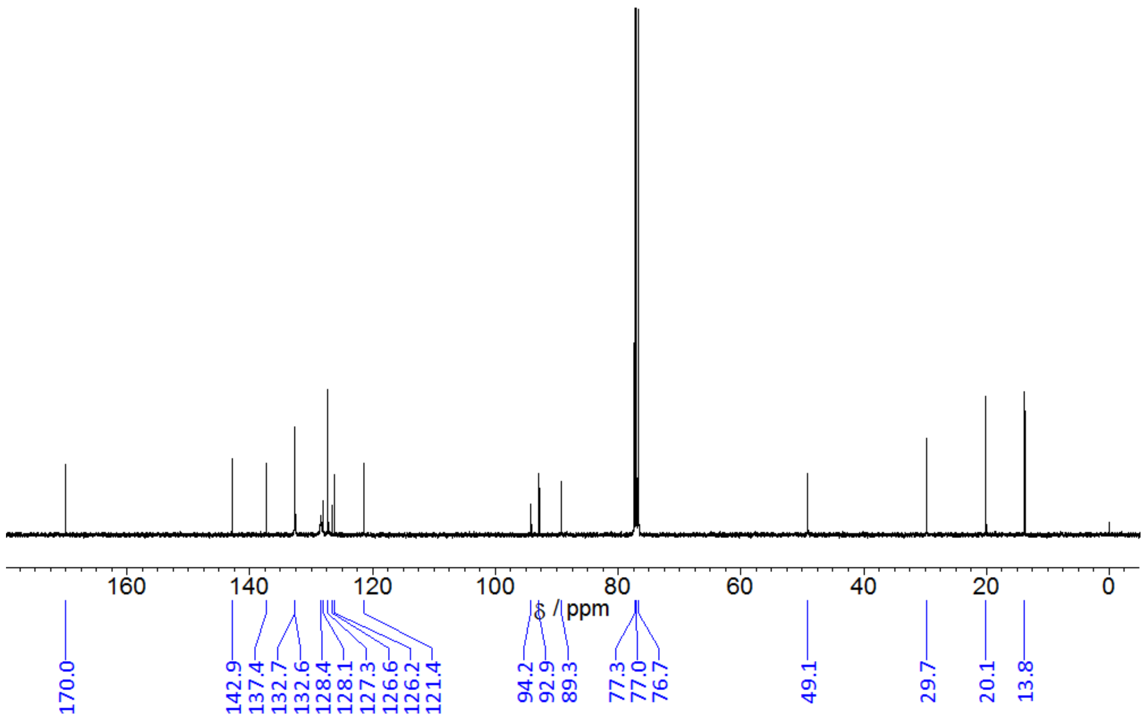

^{1}H (400 MHz, upper) and ^{13}C (100 MHz, lower) NMR spectra of **1a**, measured in $CDCl_3$ at room temperature.

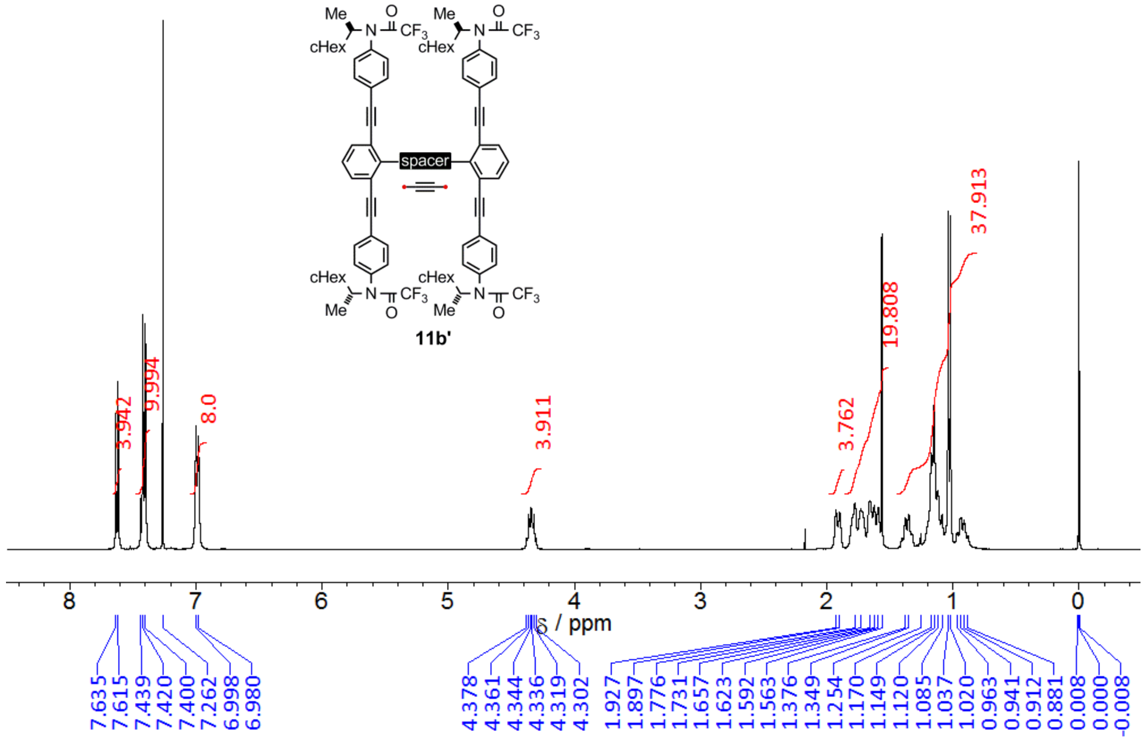

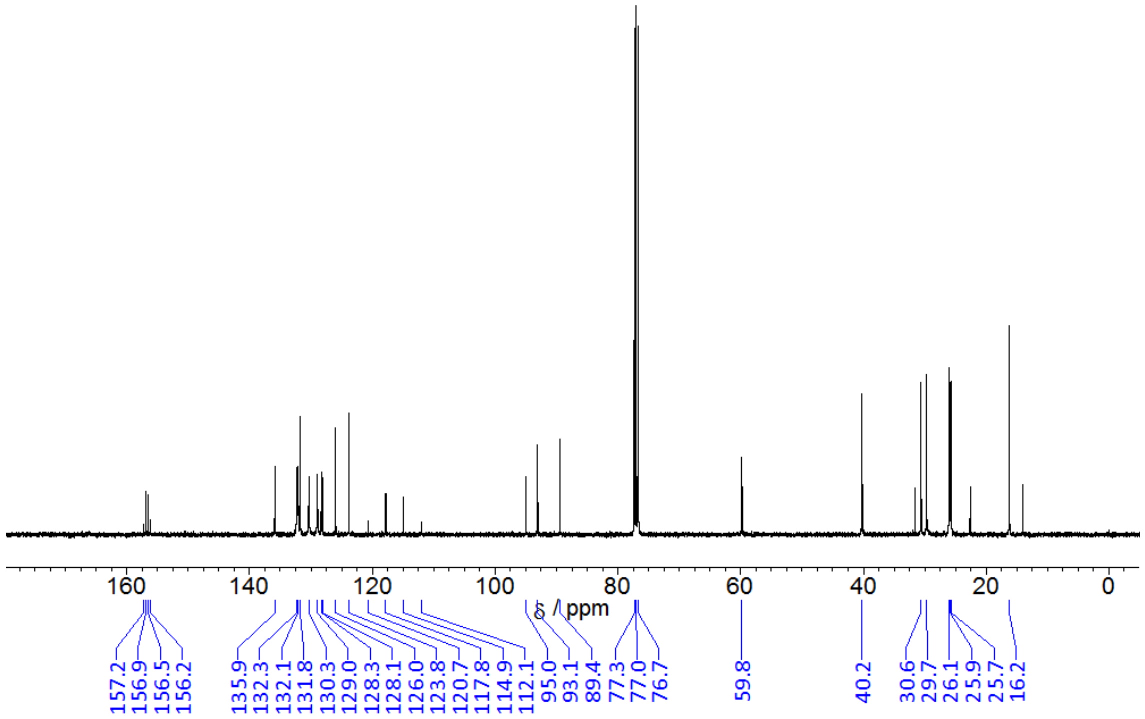

^{1}H (400 MHz, upper) and ^{13}C (100 MHz, lower) NMR spectra of **11b'**, measured in $CDCl_3$ at room temperature, cont. residual hexane in the latter spectrum.

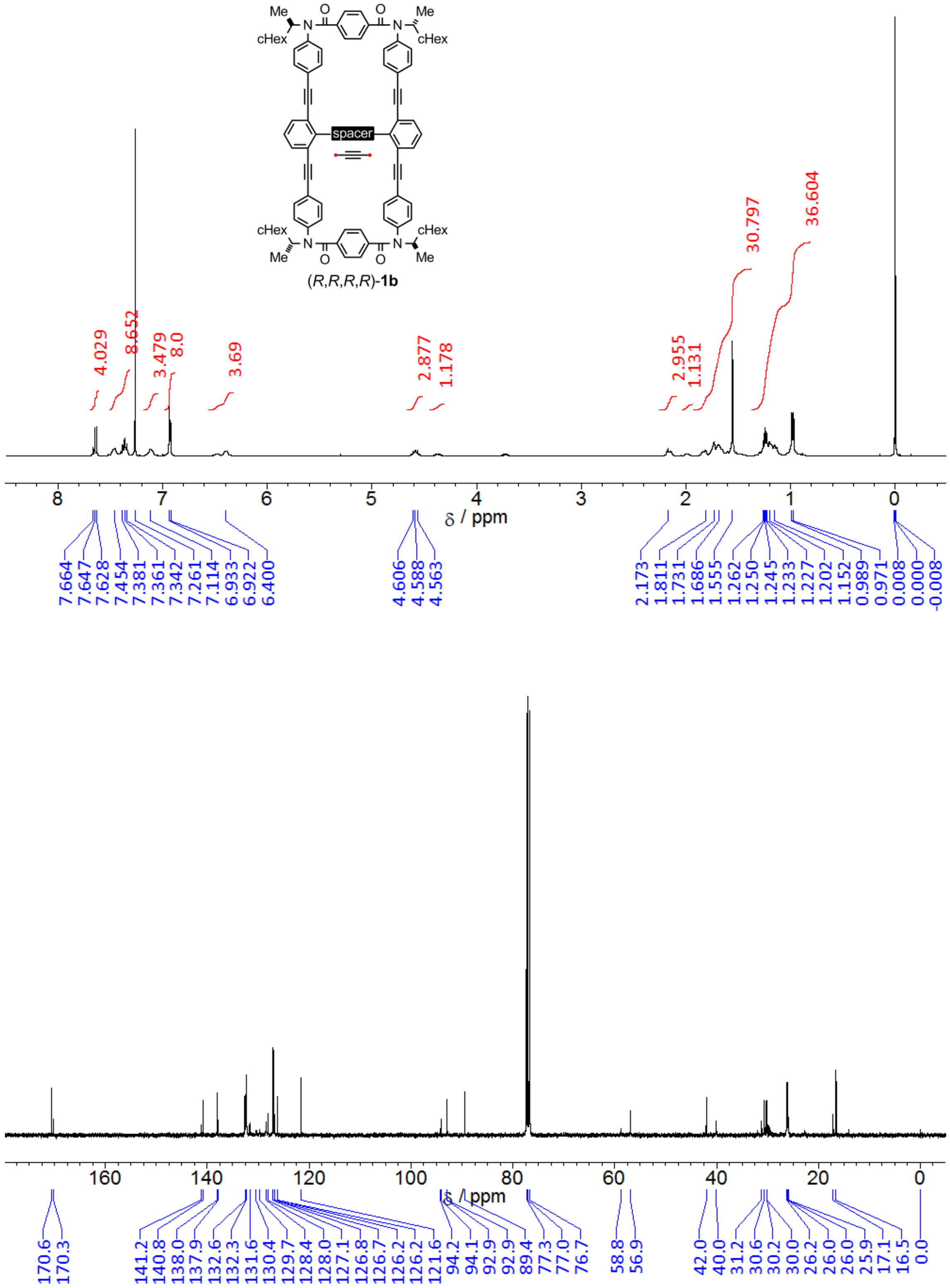

^{1}H (400 MHz, upper) and ^{13}C (100 MHz, lower) NMR spectra of (*R*,*R*,*R*,*R*)-**1b**, measured in $CDCl_3$ at room temperature, cont. residual hexane in the latter spectrum.

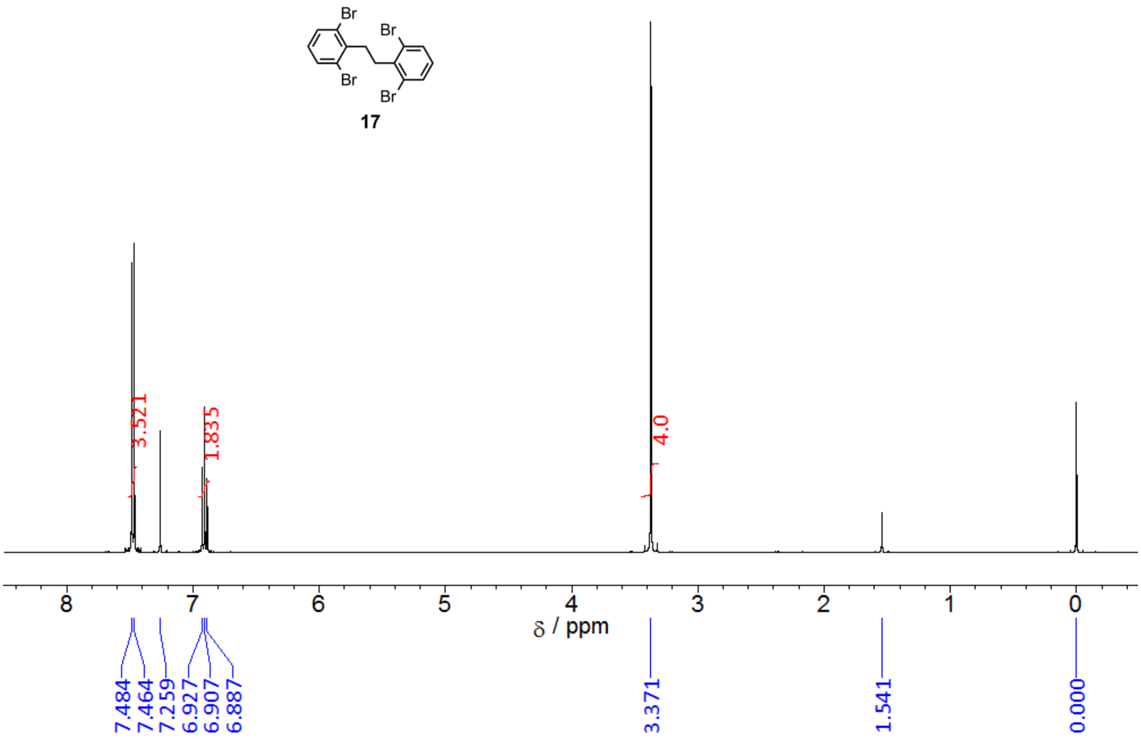

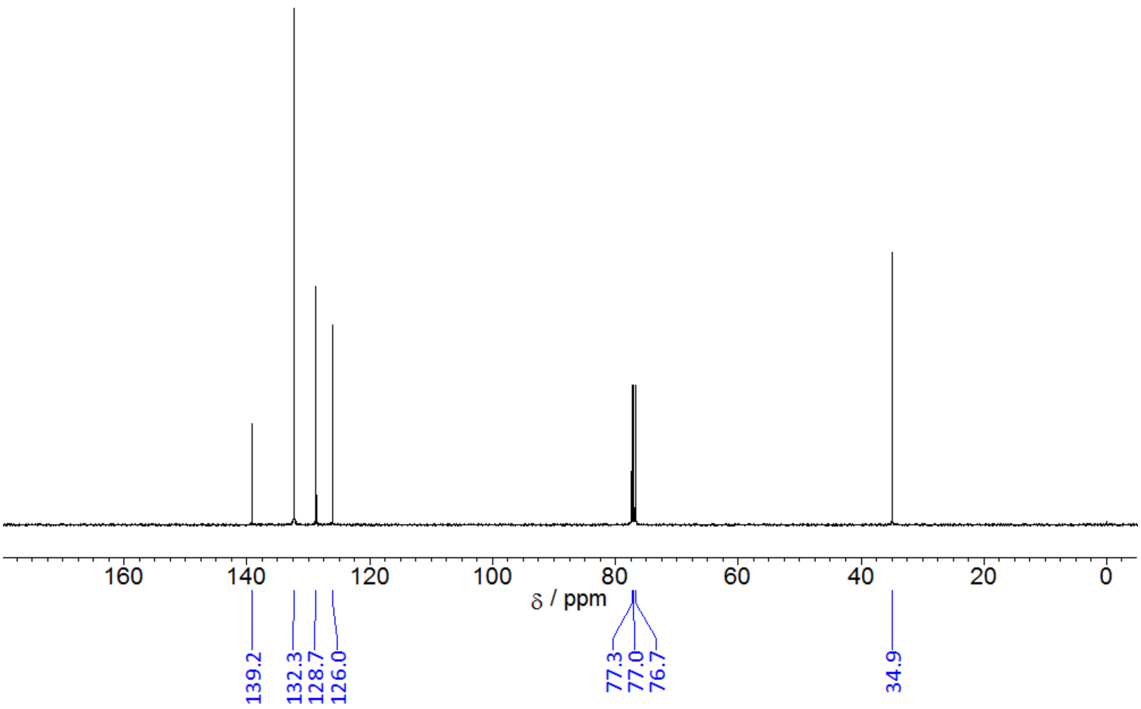

^{1}H (400 MHz, upper) and ^{13}C (100 MHz, lower) NMR spectra of **17**, measured in $CDCl_3$ at room temperature.

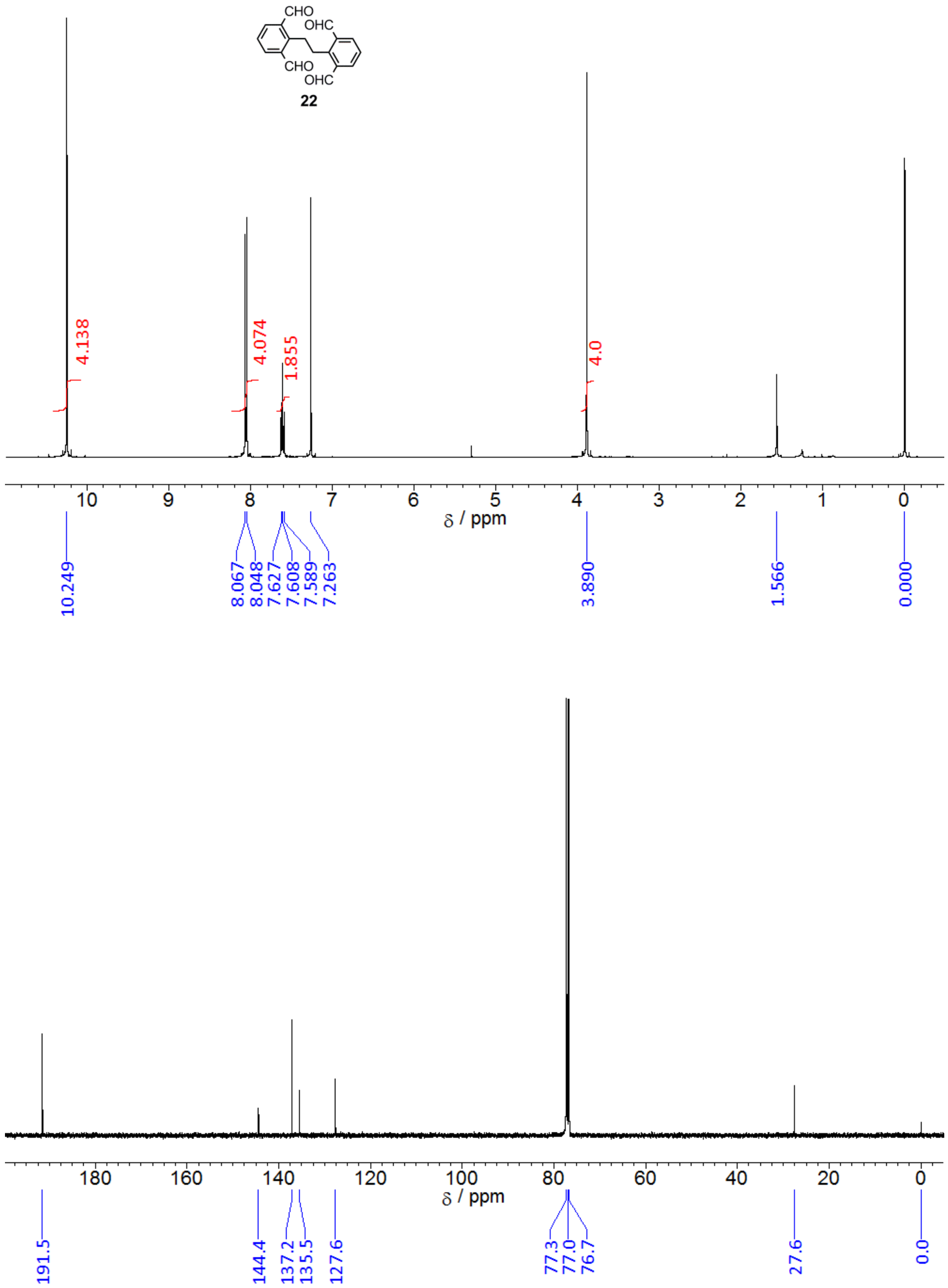

^1H (400 MHz, upper) and ^{13}C (100 MHz, lower) NMR spectra of **22**, measured in $CDCl_3$ at room temperature.

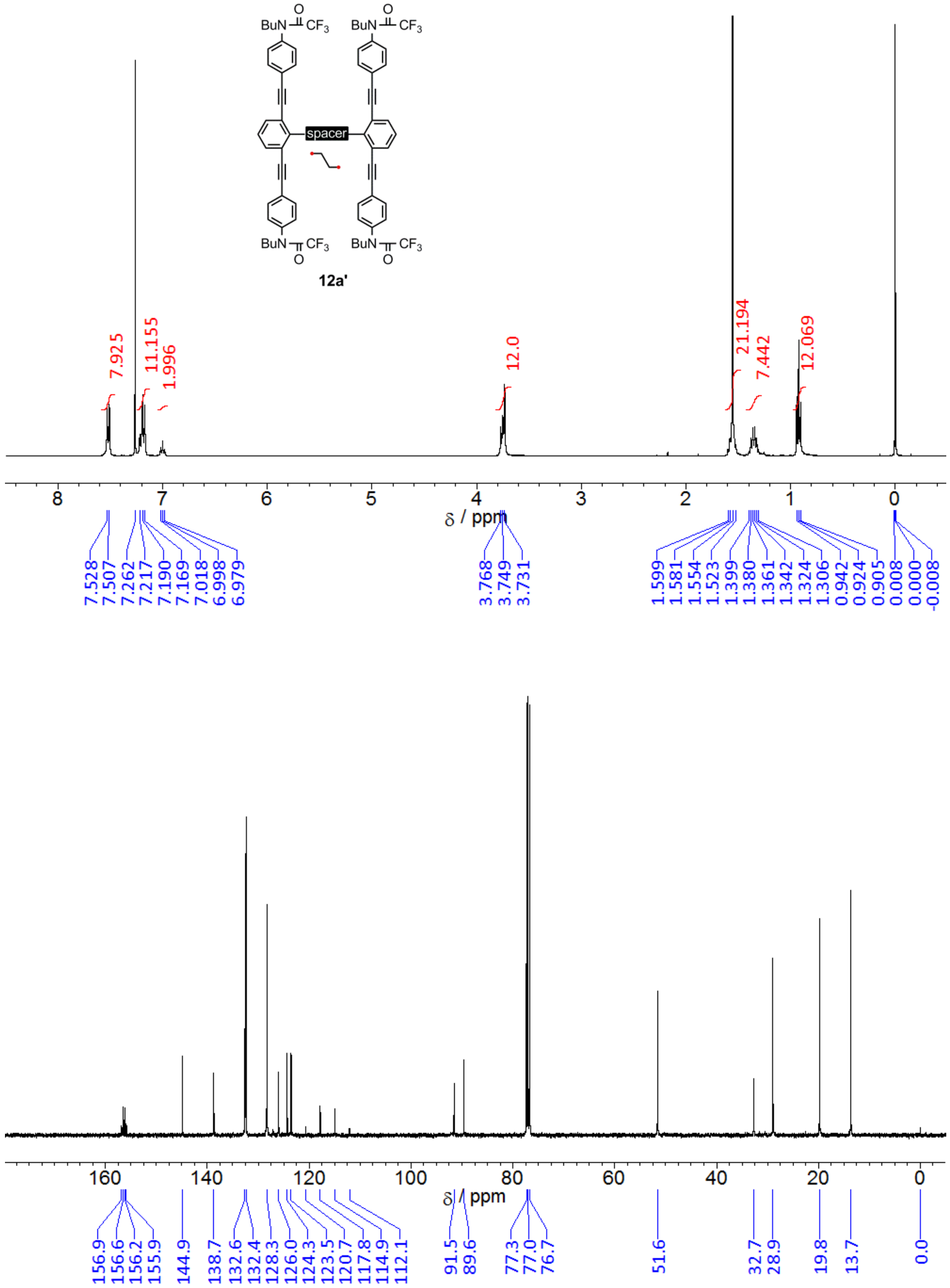

^{1}H (400 MHz, upper) and ^{13}C (100 MHz, lower) NMR spectra of **12a'**, measured in $CDCl_3$ at room temperature.

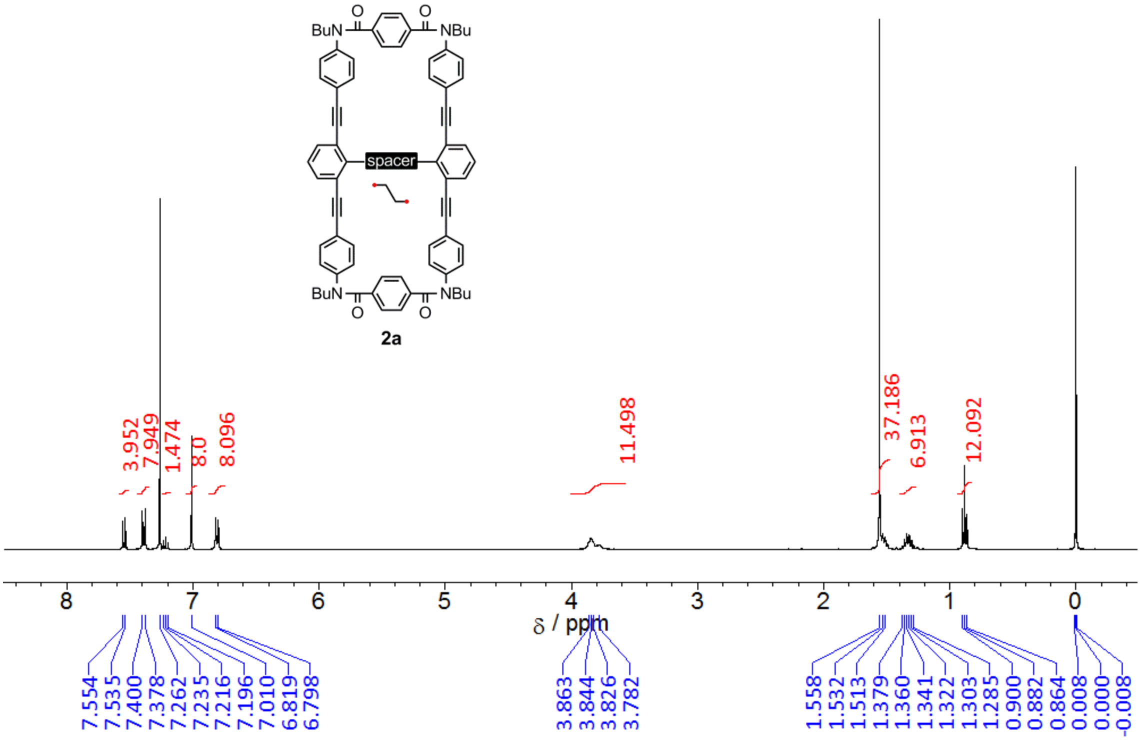

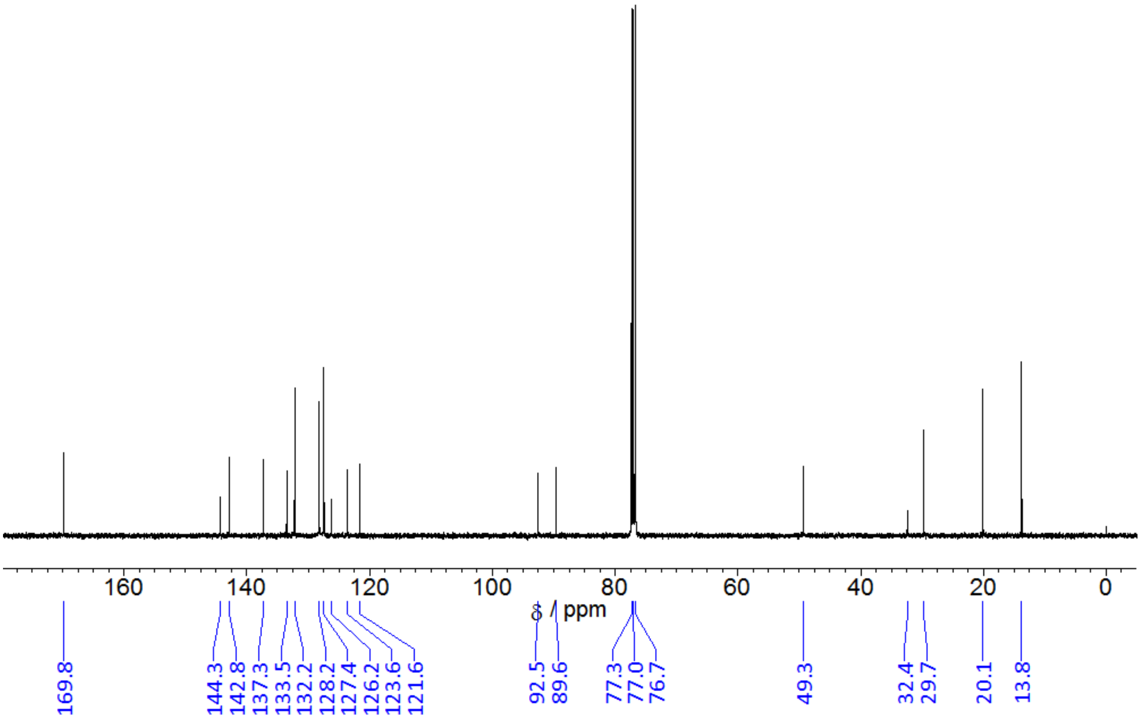

^{1}H (400 MHz, upper) and ^{13}C (100 MHz, lower) NMR spectra of **2a**, measured in $CDCl_3$ at room temperature.

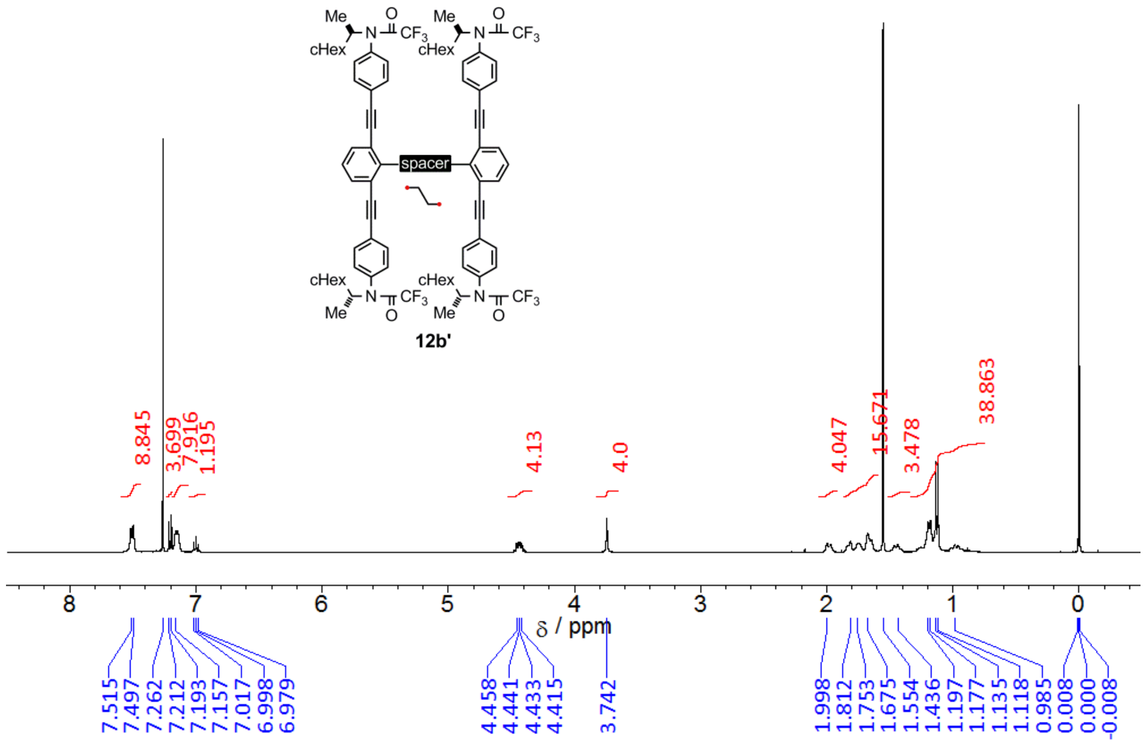

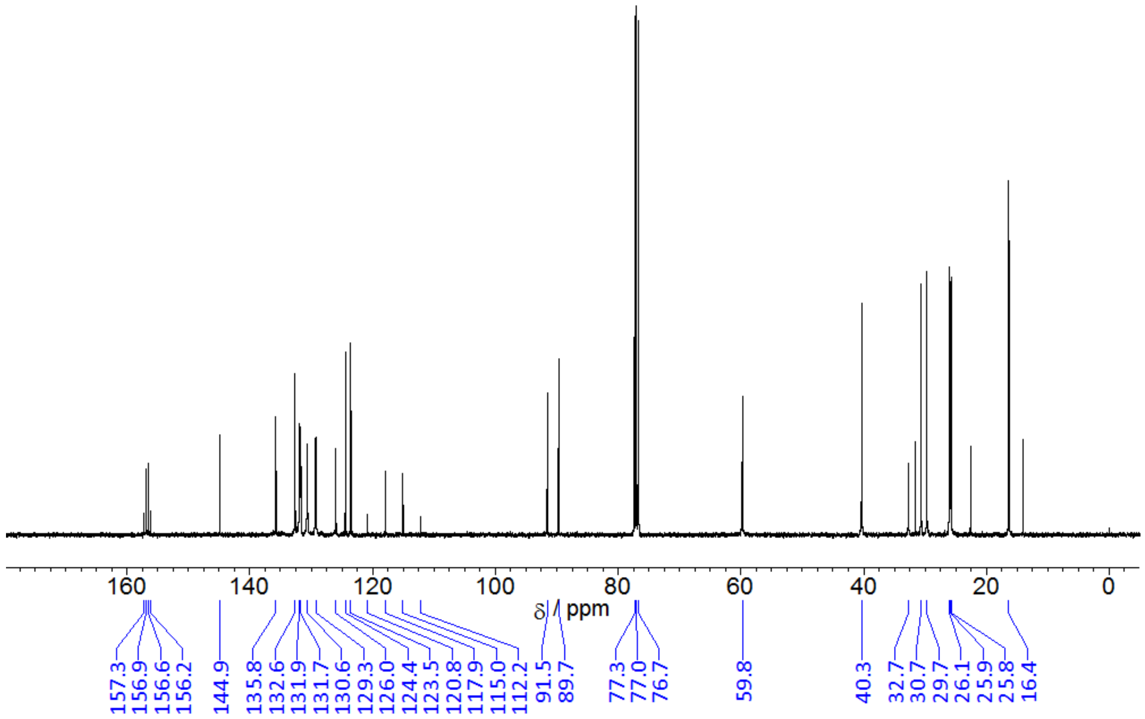

^{1}H (400 MHz, upper) and ^{13}C (100 MHz, lower) NMR spectra of **12b'**, measured in $CDCl_3$ at room temperature, cont. residual hexane in the latter spectrum.

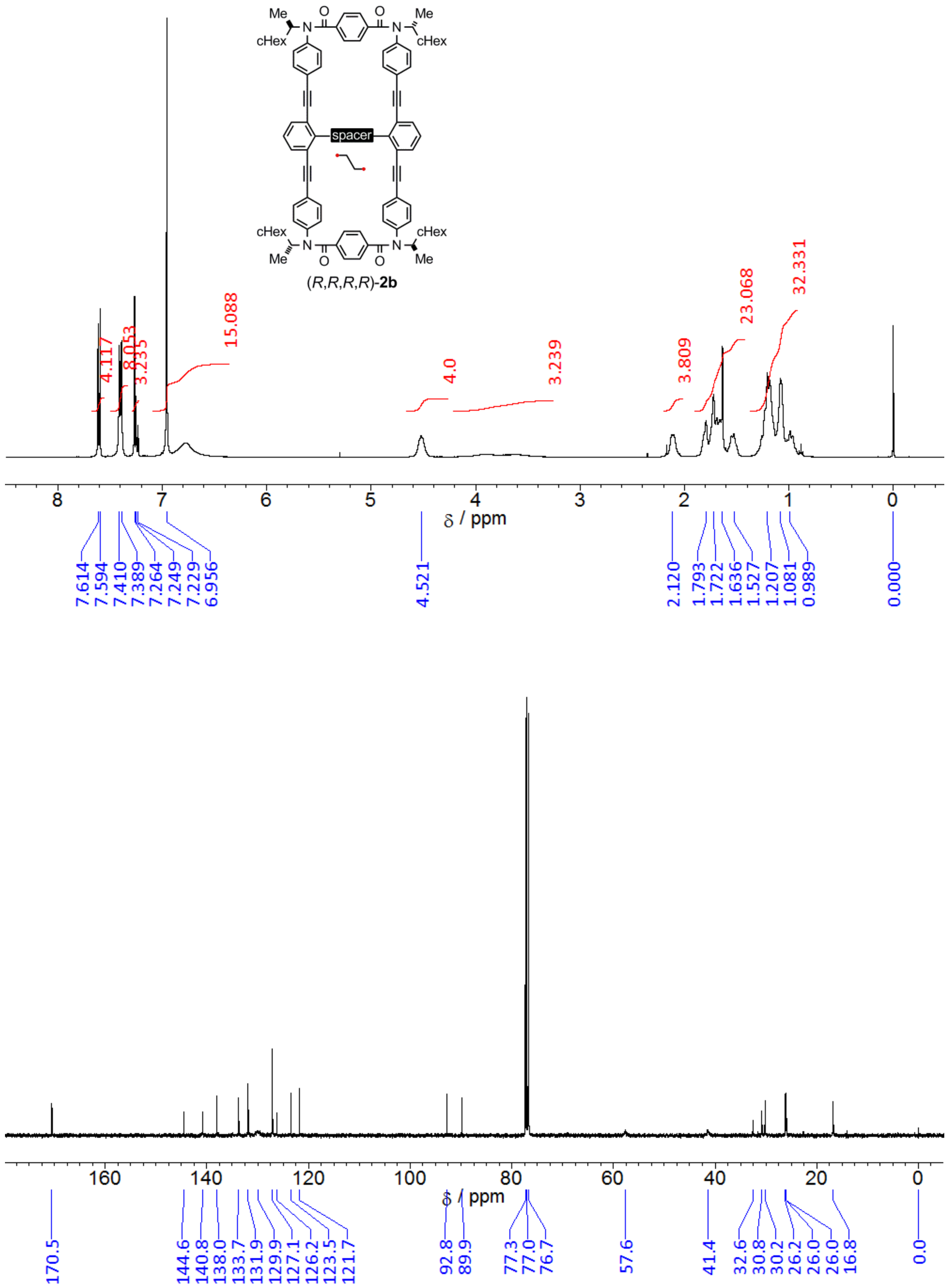

^{1}H (400 MHz, upper) and ^{13}C (100 MHz, lower) NMR spectra of (*R*,*R*,*R*,*R*)-**2b**, measured in $CDCl_3$ at room temperature.

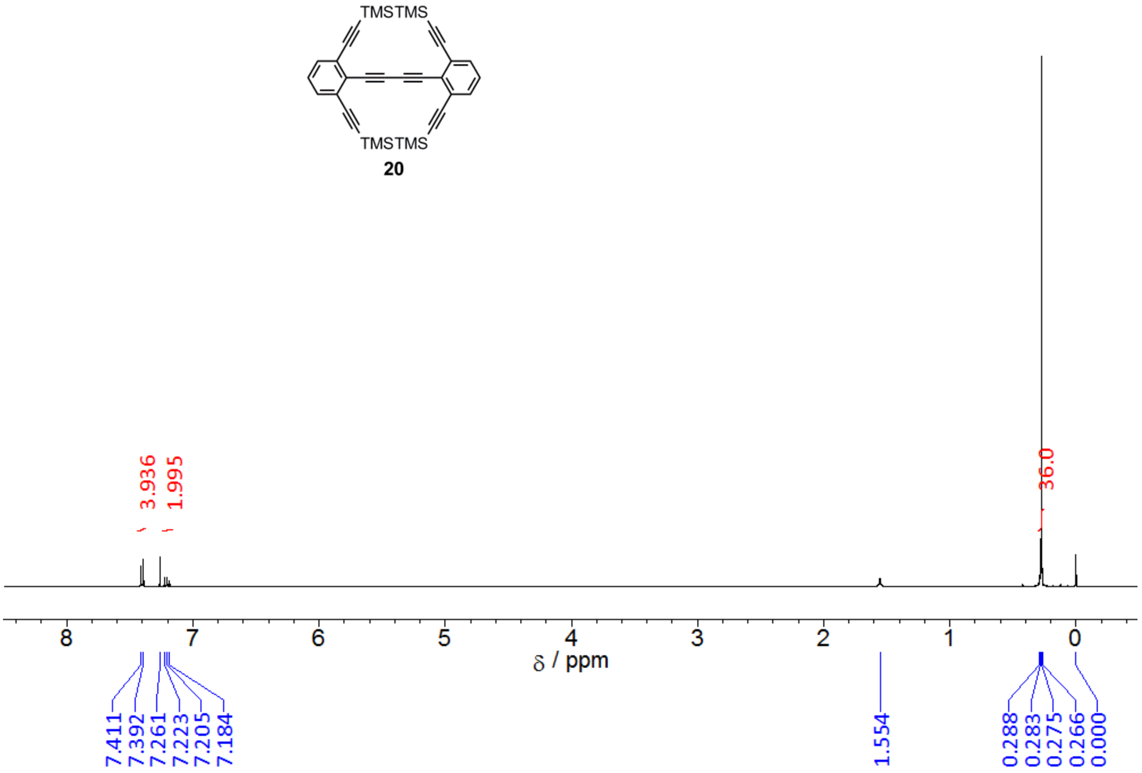

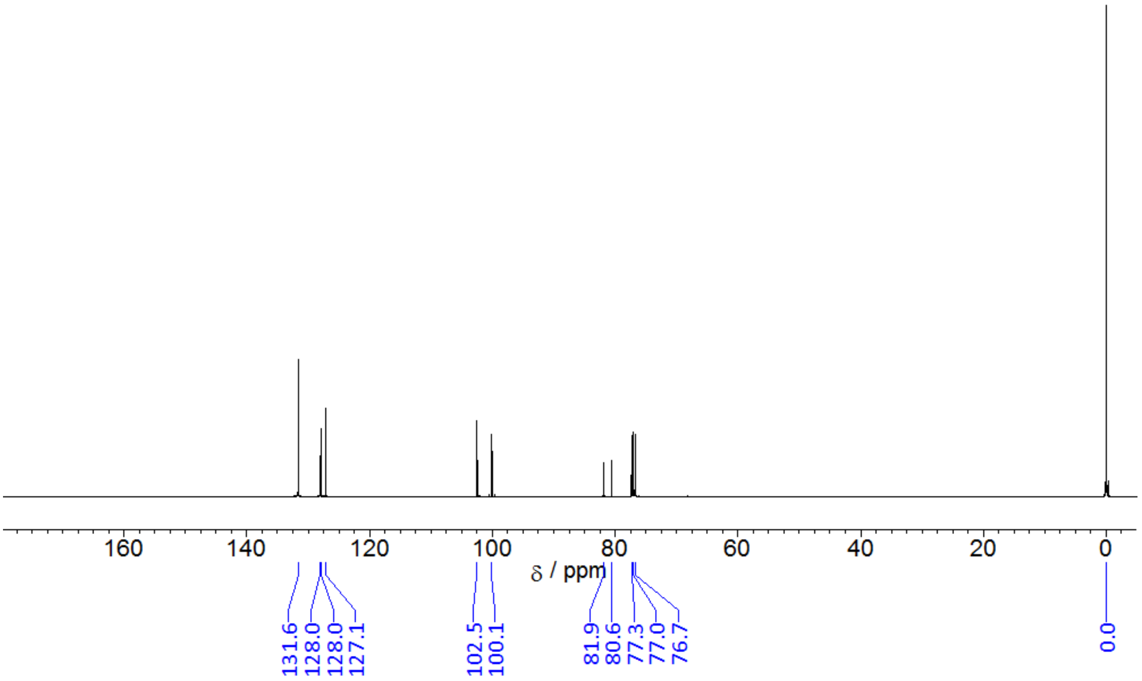

^{1}H (400 MHz, upper) and ^{13}C (100 MHz, lower) NMR spectra of **20**, measured in $CDCl_3$ at room temperature.

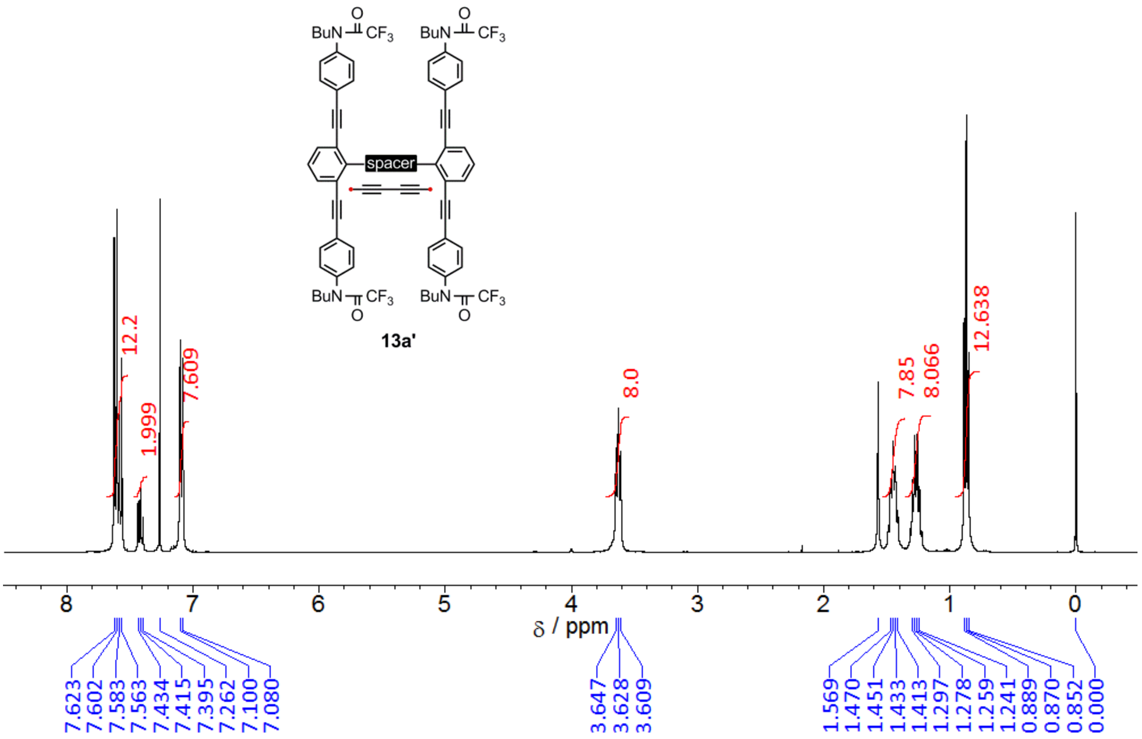

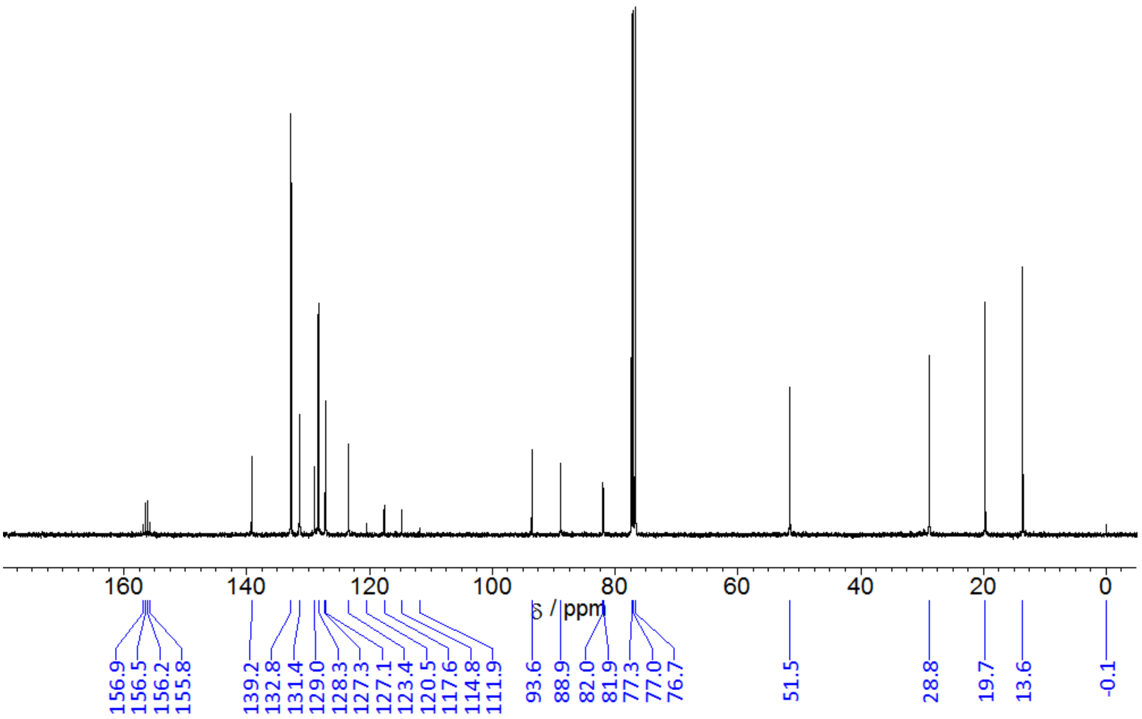

^{1}H (400 MHz, upper) and ^{13}C (100 MHz, lower) NMR spectra of **13a'**, measured in $CDCl_3$ at room temperature.

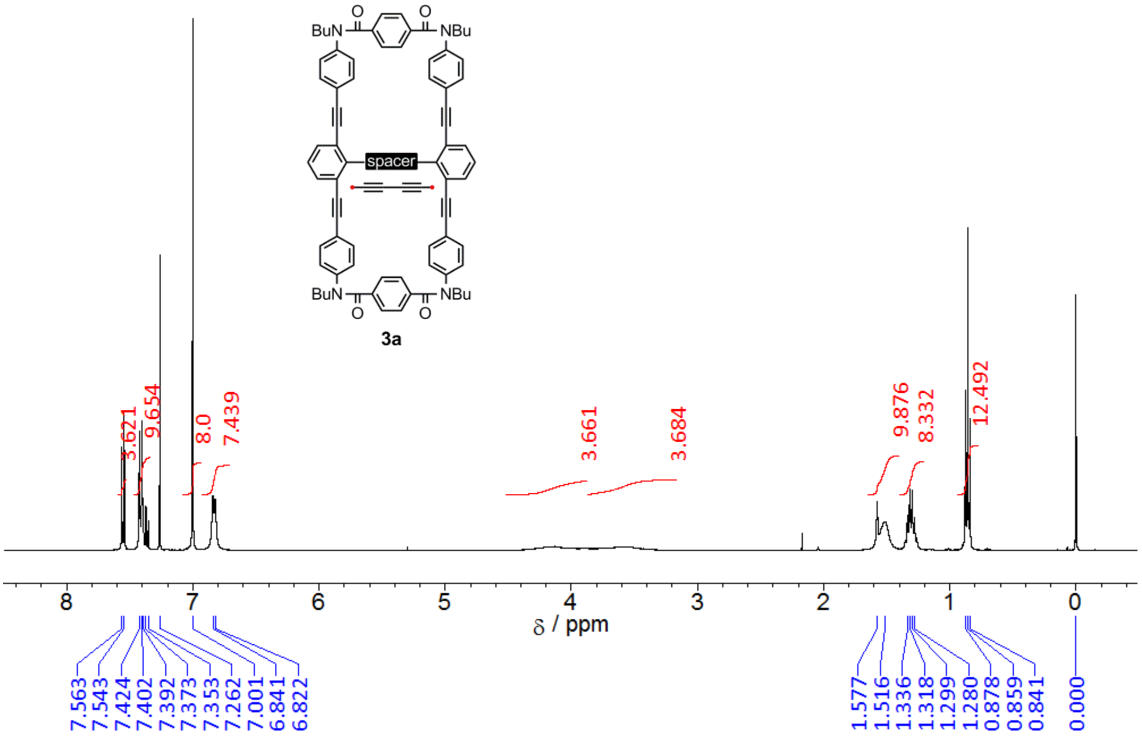

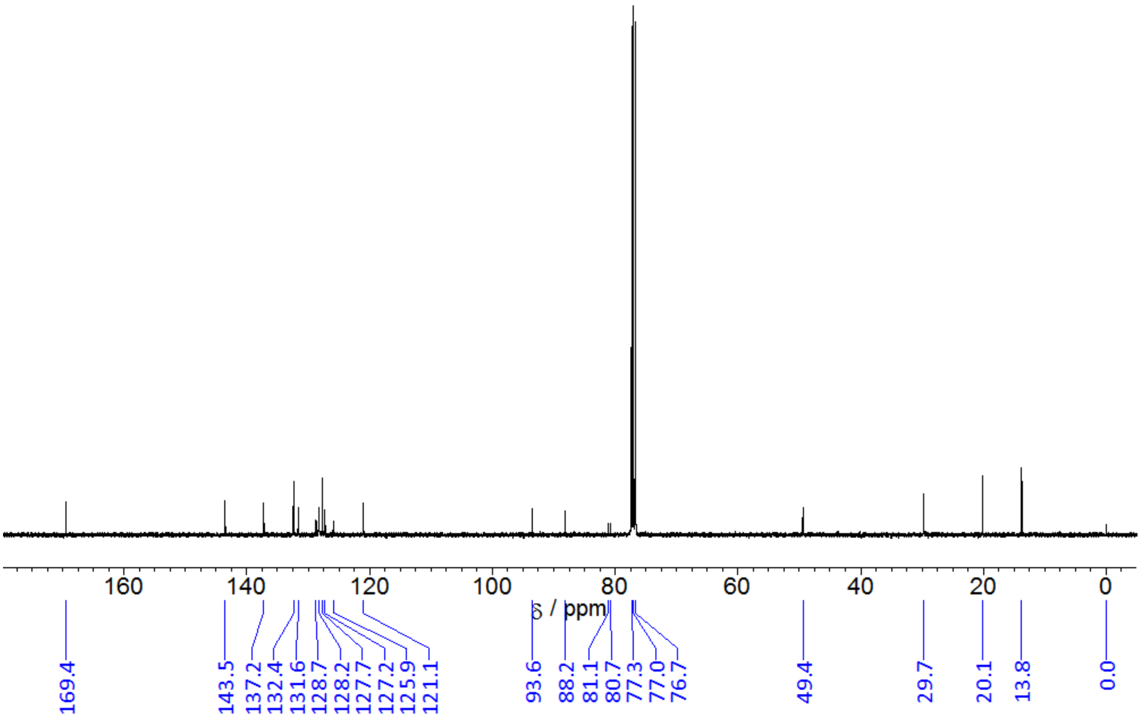

^{1}H (400 MHz, upper) and ^{13}C (100 MHz, lower) NMR spectra of **3a**, measured in $CDCl_3$ at room temperature.

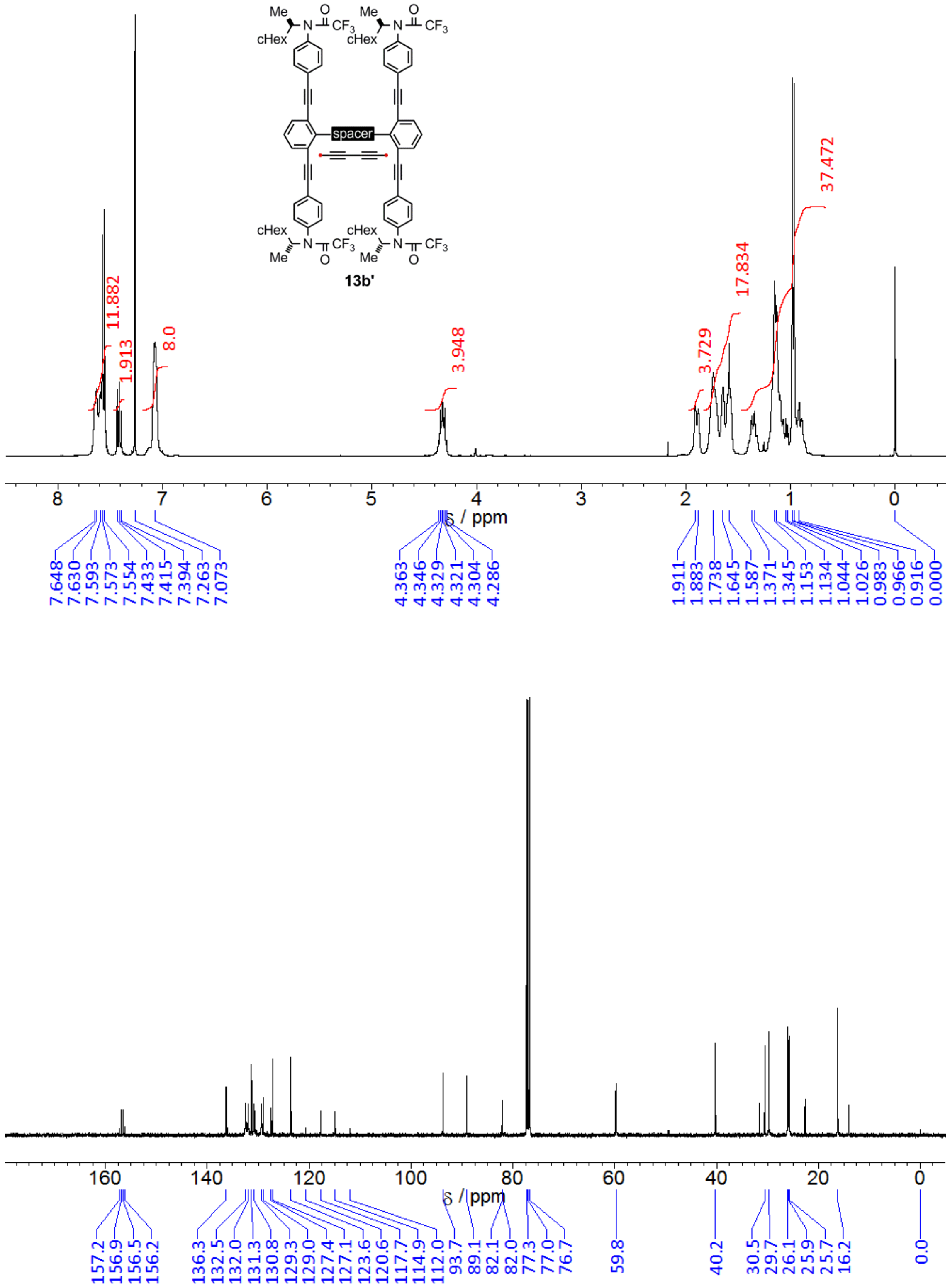

^{1}H (400 MHz, upper) and ^{13}C (100 MHz, lower) NMR spectra of **13b'**, measured in $CDCl_3$ at room temperature, cont. residual hexane in the latter spectrum.

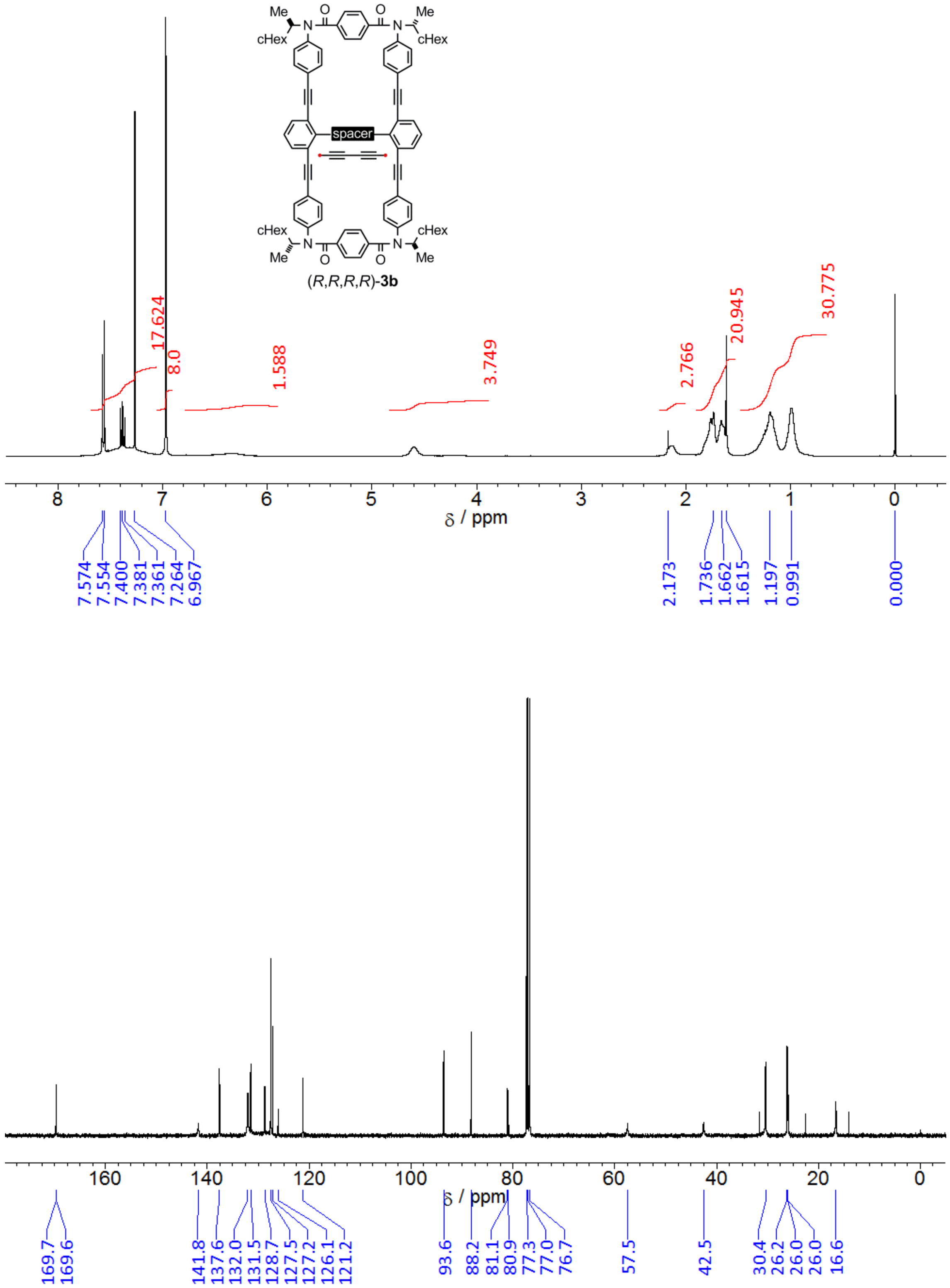

^{1}H (400 MHz, upper) and ^{13}C (100 MHz, lower) NMR spectra of (*R*,*R*,*R*,*R*)-**3b**, measured in $CDCl_3$ at room temperature, cont. residual hexane in the latter spectrum.

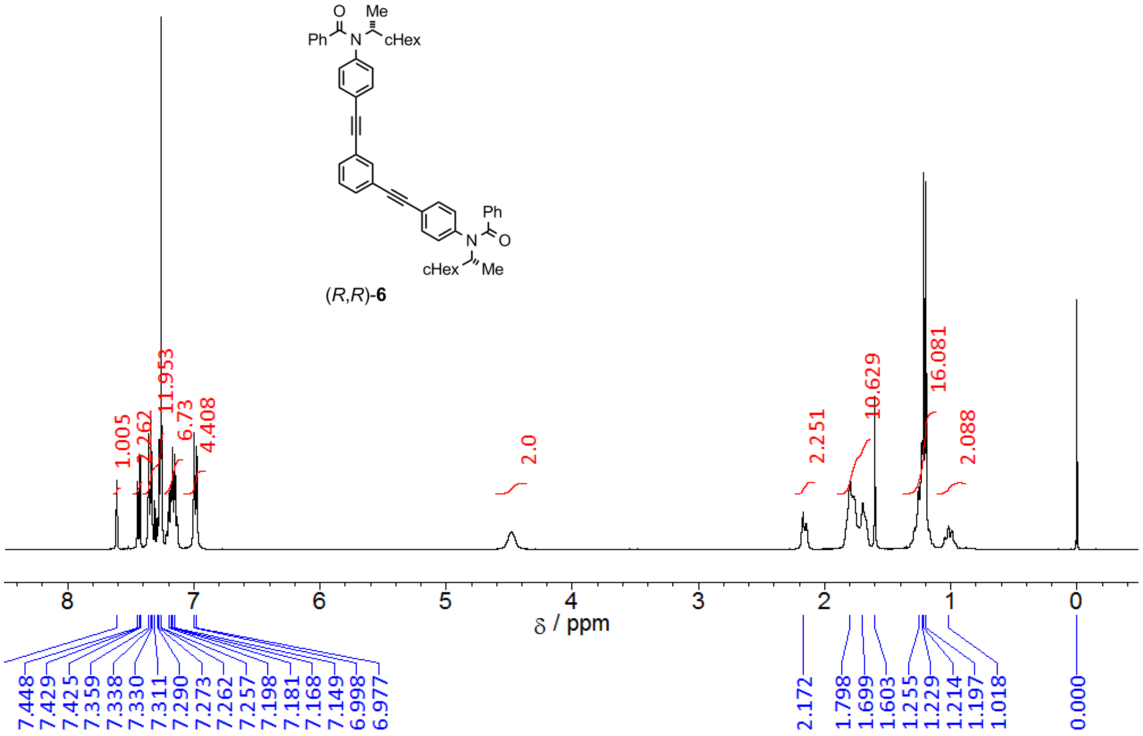

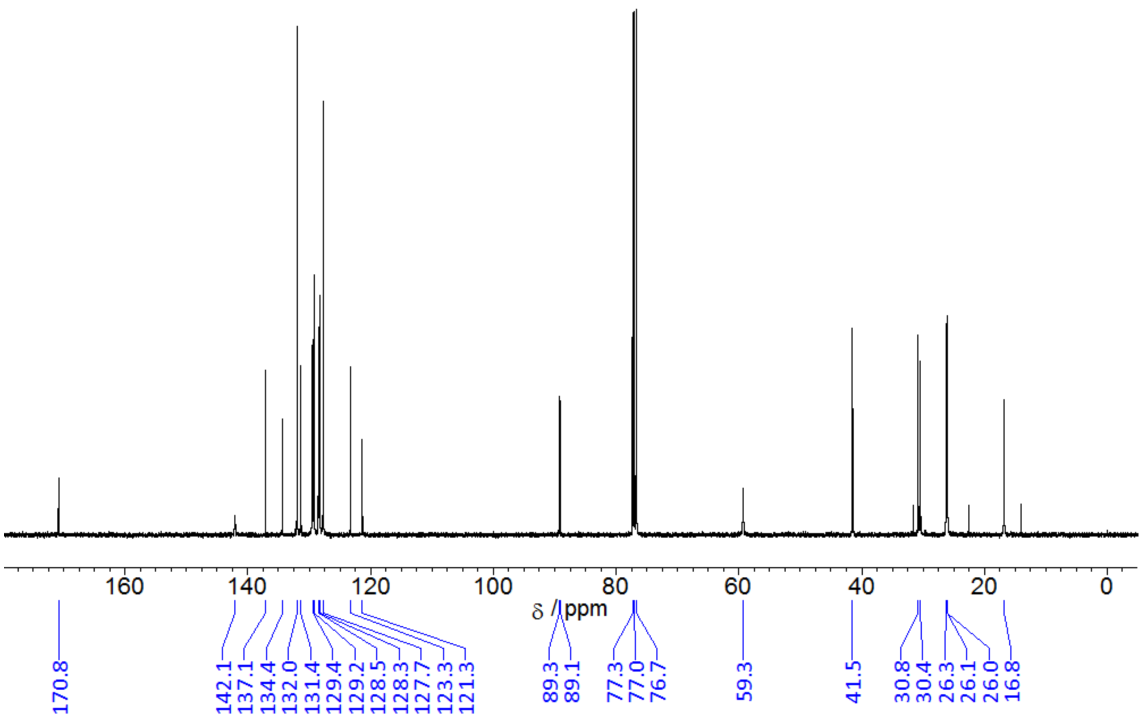

^{1}H (400 MHz, upper) and ^{13}C (100 MHz, lower) NMR spectra of (*R*,*R*)-**6**, measured in $CDCl_3$ at room temperature, cont. residual hexane in the latter spectrum.

References

1 G. Mao, A. Orita, D. Matsuo, T. Hirate, T. Iwanaga, S. Toyota, J. Otera, *Tetrahedron Lett.*, 2009, **50**, 2860–2864.

2 R. Katoono, K. Kusaka, K. Fujiwara and T. Suzuki, *Chem. Asian J.*, 2014, **9**, 3182–3187.

3 K. Sato, Y. Inoue, T. Mori, A. Sakaue, A. Tarui, M. Omote, I. Kumadaki and A. Ando, *Org. Lett.*, 2014, **16**, 3756–3759.

4 M. M. Haley, M. L. Bell, S. C. Brand, D. B. Kimball, J. J. Pak, W. B. Wan, *Tetrahedron Lett.*, 1997, **38**, 7483–7486.
